# A novel synthesis of two decades of microsatellite studies on European beech reveals decreasing genetic diversity from glacial refugia

**DOI:** 10.1007/s11295-022-01577-4

**Published:** 2022-12-12

**Authors:** Camilla Stefanini, Katalin Csilléry, Bartosz Ulaszewski, Jarosław Burczyk, Michael E. Schaepman, Meredith C. Schuman

**Affiliations:** 1 Biodiversity and Conservation Biology Unit, Swiss Federal Research Institute WSL, Zürcherstrasse 111, 8903 Birmensdorf, Dietikon, Switzerland; 2 Department of Genetics, Faculty of Biological Sciences, Kazimierz Wielki University, Chodkiewicza 30, 85-064 Bydgoszcz, Poland; 3 Remote Sensing Laboratories, Department of Geography, University of Zurich, Winterthurerstrasse 190, 8057 Zurich, Switzerland; 4 Department of Chemistry, University of Zurich, Winterthurerstrasse 190, 8057 Zurich, Switzerland

**Keywords:** Nuclear markers, Chloroplast markers, Microsatellites, Single nucleotide polymorphisms (SNPs), *Fagus sylvatica*, Genetic diversity, Demography, Heterozygosity, Min–max scaling

## Abstract

Genetic diversity influences the evolutionary potential of forest trees under changing environmental conditions, thus indirectly the ecosystem services that forests provide. European beech (*Fagus sylvatica* L.) is a dominant European forest tree species that increasingly suffers from climate change-related die-back. Here, we conducted a systematic literature review of neutral genetic diversity in European beech and created a meta-data set of expected heterozygosity (*He*) from all past studies providing nuclear microsatellite data. We propose a novel approach, based on population genetic theory and a min–max scaling to make past studies comparable. Using a new microsatellite data set with unprecedented geographic coverage and various re-sampling schemes to mimic common sampling biases, we show the potential and limitations of the scaling approach. The scaled meta-dataset reveals the expected trend of decreasing genetic diversity from glacial refugia across the species range and also supports the hypothesis that different lineages met and admixed north of the European mountain ranges. As a result, we present a map of genetic diversity across the range of European beech which could help to identify seed source populations harboring greater diversity and guide sampling strategies for future genome-wide and functional investigations of genetic variation. Our approach illustrates how to combine information from several nuclear microsatellite data sets to describe patterns of genetic diversity extending beyond the geographic scale or mean number of loci used in each individual study, and thus is a proof-of-concept for synthesizing knowledge from existing studies also in other species.

**Supplementary Information:**

The online version contains supplementary material available at 10.1007/s11295-022-01577-4.

## Introduction

In 1988, Paul Ehrlich predicted that the loss of genetic diversity was already “at least as important a problem as the loss of entire species” (p. 22) and that this loss would cause a “disruption of the course of evolution, insofar as speciation processes will have to work with a greatly reduced pool of species and their genetic materials” (p. 32, Wilson and Frances, eds. 1988). Indeed, today, there is abundant evidence that forest tree populations harboring lower genetic diversity suffer negative fitness consequences and compromised disease resistance (Aerts et al. 2011; Smulders et al. 2008; Zeng et al. 2021). The role of genetic markers in conservation biology started with the advent of isozyme markers in the 1970s, which first permitted demonstration of the high within-population genetic diversity of many forest tree species (e.g., Lander et al. 2011; Pluess 2011; Shi and Chen 2012; Lesser et al. 2013; Elleouet and Aitken 2019), even in rare endemic species (Conkle 1992).

Fifty years later, geneticists continue to develop higher-throughput, increasingly transferable methods to facilitate the study of genetic diversity in and across populations (Metzker 2010; Nekrutenko and Taylor 2012). The most abundant information about genetic diversity generally concerns neutral genetic diversity (i.e., loci that are not under selection), because neutral markers, such as isozymes and microsatellites, were developed earlier and have been longer in use. Although its use as a metric to assess populations is increasingly debated (Teixeira and Huber 2021), the analysis of neutral loci provides valuable information about key processes in ecology and evolution, such as migration patterns and kinship. Studies generally assess genetic variation for a set of populations from a systematic but sparse sampling of individuals, which, in turn, often represent a small portion of a species range. The synthesis of genetic records generated across many studies and over decades, therefore, represents a precious source of information for understanding the genetic structure of a species and how it may be evolving as a result of demographic and environmental effects.

*Fagus sylvatica* L. is a broadleaf tree species widespread throughout Europe, ranging from the southern Mediterranean to Scandinavia in the north, where the cold climate limits its distribution (Fang et al. 2006; Bolte et al. 2007), and spanning from the north-eastern Iberian Peninsula in the west, to the Carpathian Mountains in the east (Caudullo et al. 2017). As the dominant tree species in many European low mountain and lowland forest ecosystems (Rose et al. 2009), *F. sylvatica* has great ecological importance, in addition to its economic value as a source of wood. The geographic distribution of *F. sylvatica* is likely limited by its sensitivity to drought (Aranda et al. 2015; Cuervo-Alarcon et al. 2021) and late frost (Dittmar and Elling 2006; Kreyling et al. 2012; Sangüesa-Barreda et al. 2021), although some studies indicate that *F. sylvatica* is more resilient to these factors compared to other European forest species, such as spruce and larch (Príncipe et al. 2017; Vitasse et al. 2019), and populations on the margins of the species range may harbor more resistance to drought (Muffler et al. 2020). Common garden experiments with two Greek beech provenances from the southeastern edge of the species range revealed phenological plasticity in response to varying temperature and precipitation (Varsamis et al. 2019). Studies based on species distribution models (SDMs) predict that the potential range of *F. sylvatica* will expand toward north-eastern Europe and to higher elevations, accompanied by a gradual range reduction in the south, under different future climate scenarios (Fang and Lechowicz 2006; Silva et al. 2012; Falk and Hempelmann 2013; Duputié et al. 2015; Saltré et al. 2015; Dyderski et al. 2018; Jiménez-Alfaro et al. 2018; Varsamis et al. 2019; Capblancq et al. 2020a). Yet drought and frost events, together with deforestation and expanding human land use, are expected to contribute to the significant decline in actual *F. sylvatica* populations (Sjolund et al. 2017). Therefore, the future distribution of European beech forests under climate change is unclear (Brun et al. 2020; Schuldt et al. 2020; Pfenninger et al. 2021).

For most temperate forest trees, including *F. sylvatica*, the spatial distribution of genetic diversity has been shaped by multiple range contraction and expansion events during repeated glacial and inter-glacial cycles (Magri et al. 2006). Based on chloroplast and nuclear marker data (isozymes) combined with fossil records, Magri et al. (2006) suggested that central and northern Europe were colonized from a single or few refugia situated on the Balkan peninsula after the last glacial maximum (LGM), while other supposed Mediterranean refugia, located in the Italian and Iberian peninsulas, likely did not contribute to the post-LGM range expansion. Other studies based on nuclear microsatellite markers sampled smaller portions of the species range, but indicated the presence of local microrefugia in the southern Balkans, in northern Italy and the northern Alps, in Greece, and in the Pyrenees (Emiliani et al. 2004; Giesecke et al. 2007; Magri 2008; De Lafontaine et al. 2013; Saltré et al. 2013), and putative multiple subsequent expansion events, adding complexity to the demographic history of *F. sylvatica*. Thus, beech might have expanded from these multiple refugia through several colonization routes toward central and northern Europe and possibly admixed in regions along the colonization front (Magri 2008; Kempf et al. 2016; Lander et al. 2021).

Due to the large effective population size of open-pollinated forest trees, the rate of change in genetic diversity is slow and scales with 2N_e_ generations (Ellegren and Galtier 2016). Thus, most contemporary populations should have similar levels of genetic diversity, reflecting the effective population size of the refugial population. Such spatially homogeneous patterns of diversity, along with a weak population divergence, have been identified in European beech. Nevertheless, several mechanisms could contribute to local heterogeneity. First, regions at the colonization front, where different lineages are admixed, could also represent genetic diversity hot spots, as has been observed for tree species north of the Alps (Petit et al. 2003). Second, during range expansion in forest trees, genetic diversity is often lost due to repeated founder events (Excoffier et al. 2009), counterbalanced by long-distance pollen flow that tends to maintain diversity (e.g., Wang et al. 2016; Lander et al. 2021; Postolache et al. 2021), such that the current distribution is influenced by the balance of these two factors. Thus, it is risky to attempt to project range-wide genetic diversity from studies which do not cover the entire species range, as partially opposing and spatially heterogeneous processes are likely to be relevant. Despite the important advances represented by individual studies, these are usually geographically limited and employ different markers and marker systems, making range-wide comparisons challenging.

An important solution to the problem of different marker systems, as well as the need to select a priori to study either neutral or functional genetic diversity, is on the horizon in the form of cost-effective whole-genome sequencing and sequence analysis to identify single-nucleotide polymorphisms (SNPs) and other genomic markers. Reference nuclear (Mishra et al. 2022), plastid (Mader et al. 2019; Mishra et al. 2021b), and mitochondrial (Mishra et al. 2021a) genomes of *F. sylvatica* have recently been published. Yet, the currently employed methods for cost-effective SNP identification, such as restriction-site-associated DNA (RAD) sequencing (Peterson et al. 2012), tend to bias results toward neutral loci and have high missing data rates. However, compiling data from the many already-available microsatellite-based studies is still a valuable option for inferring range-wide genetic diversity patterns of species.

Here, we consolidate current knowledge on the genetic diversity of *F. sylvatica* from more than three decades of marker-based studies, depict overall coverage of the species’ natural range, identify gaps, and place these in the context of newer SNP-based studies. Specifically, we (1) summarize literature on the genetic diversity of *F. sylvatica* in terms of geographic distribution, numbers of studies and studied populations, sample sizes, and molecular marker systems; (2) combine available data from the most abundantly used marker system, nuclear microsatellites, to compile a meta-dataset; (3) apply a scaling approach to the meta-dataset to describe spatial genetic diversity patterns across the species range; and (4) furthermore, include a novel microsatellite data set with unprecedented geographic coverage, for which we also use various re-sampling schemes to mimic common sampling biases and show the potential and limitations of the scaling approach.

## Materials and methods

### Literature review and article assessment procedure

We followed the guidelines for systematic literature review provided by Pullin and Stewart (Pullin et al. 2006) and conducted a keyword literature search combined with a snowball approach, which included recommendations from experts. We searched PubMed, Google Scholar, ScienceDirect, Scopus, and the *Fagus sylvatica* section of the database Dryad, to identify potentially relevant publications from peer-reviewed journals and/or additional datasets. The following keywords were used: (“*Fagus sylvatica*” OR “European beech”) and (“geographic distribution,” “distribution range,” “genetic varia*,” “genetic divers*,” “environmental stress response,” “genomic differences,” or “genomic varia*”). Additional papers were identified by topical suggestions from Mendeley, which was used to keep, track, and organize the bibliography (https://www.mendeley.com/), and 75 articles were received by a subject expert (E. Magnanou, IR CNRS). Reference lists from each article were examined for additional suitable papers, which were then included in the database. In addition, publications by the first and last authors of all initially selected articles were searched on PubMed. Relevant publications were initially retained based on title assessment, and the search was conducted until no new relevant publications were identified, which resulted in 123 publications in addition to the 75 from expert recommendation (198 publications total). Four duplicates were then removed, and abstracts, tables, and figures were screened according to the list of criteria below, which excluded an additional 29 publications. The full text of the remaining 165 publications was assessed based on the same criteria as used for the initial screening:

1. published in a peer-reviewed academic journal
2. reporting on the genetics of *Fagus sylvatica* L.
3. including empirical studies with data collected from field sampling (theoretical papers were excluded from the analysis, as were simulation studies, reviews of existing data, and studies conducted in controlled environments or where genetic diversity was experimentally manipulated)
4. reported allelic richness or genetic diversity (e.g., expected heterozygosity) estimates
5. publication language confined to English

**Fig. 1:**
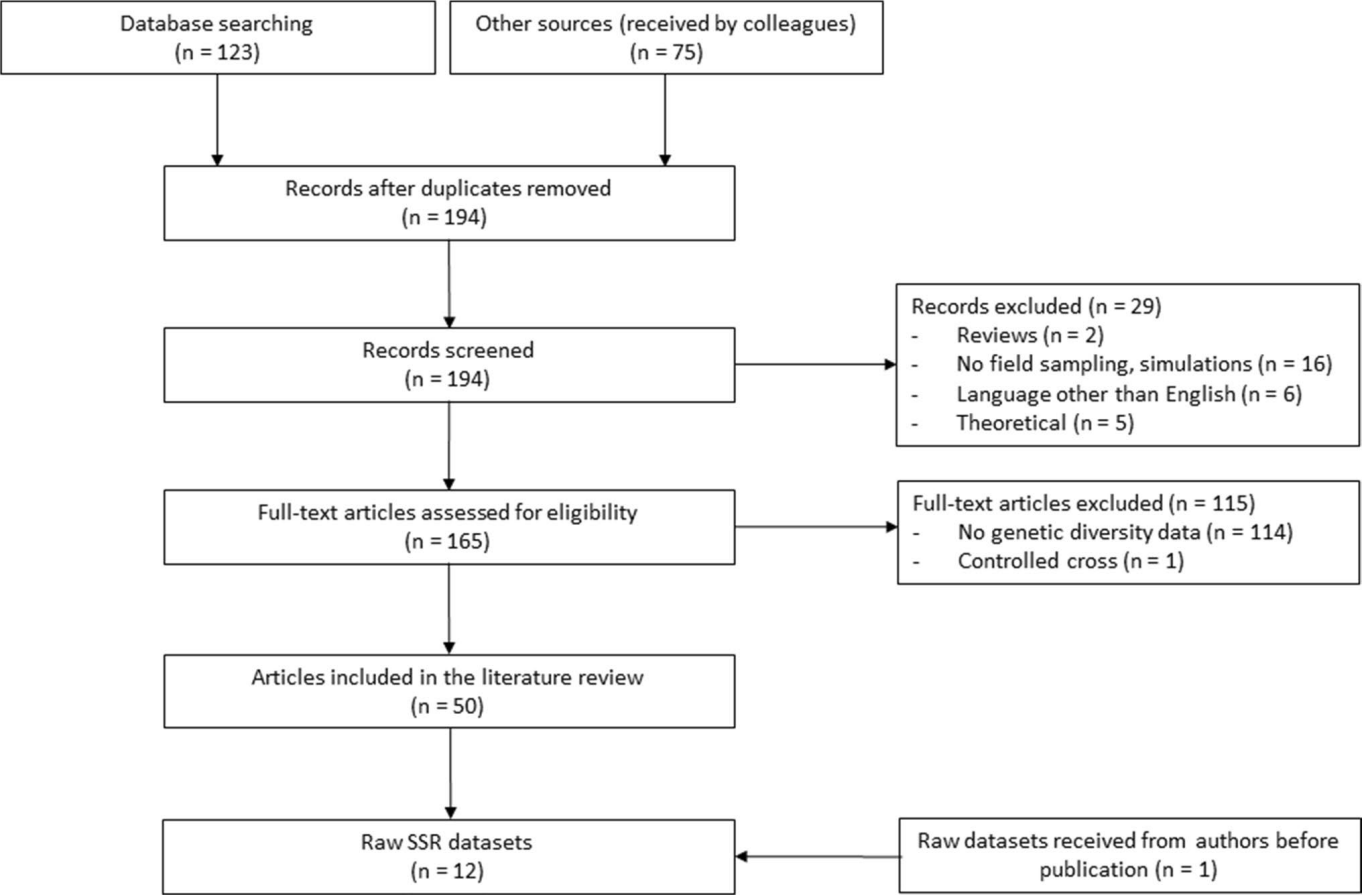
PRISMA flow diagram representing the different phases of the systematic literature review of genetic diversity in *Fagus sylvatica*

At this initial phase, studies targeting both neutral and adaptive genetic diversity (measured using molecular markers) were accepted. The final dataset comprised 50 articles published between 1982 and 2020 (Fig. 1, Table S1), after excluding 115 papers that did not meet our inclusion criteria (Table S2).

### Literature search

The source data originated from the 50 publications selected during the literature review described above and from one previously unpublished nuclear microsatellite dataset (see “Generation of additional nuclear microsatellite data”). Data from published studies were extracted directly from PDFs as described below. Additionally, raw datasets of genotyped trees were retrieved from the Dryad database for 11 publications (Lander et al. 2011; Jump et al. 2012; Lefèvre et al. 2012; Stefano et al. 2012; Piotti et al. 2012; De Lafontaine et al. 2013; Gauzere et al. 2013; Rajendra et al. 2014; Sjölund and Jump 2015; Müller et al. 2018; Oddou-Muratorio et al. 2018). An additional four datasets were obtained from the authors (Magri 2008; Sjölund et al. 2015; Cvrčková et al. 2017; Cuervo-Alarcon et al. 2018). Compiled data used for the analysis are available with the Dryad dataset for this paper, and an overview is provided in Table S1.

### Data extraction

When the source dataset was not available, data were extracted from the PDF text or from tables using the R package tabulizer (Leeper 2018) or the Tabula software (https://tabula.technology/).

The following information was extracted and tabulated from each eligible study:

• name of the first author
• year of publication
• country or countries of the sampled population(s)
• name and location of the sampled population(s) (coordinates and altitude where available)
• year of sample collection
• number of populations studied
• number of sampled individuals as a measure of sampling size
• stand age
• biological sample/type of specimen
• plot size (km2)
• molecular marker
• number of loci
• identity of loci
• proxies of genetic diversity including allelic diversity (*Na*), allelic richness (*Ar*), and the standard sample size used for the rarefaction, where reported; expected heterozygosity (*He*), and nucleotide diversity (π) or haplotype diversity (*Hd*) for the studies based on dominant markers (cpDNA)
• global fixation index (*Fst*) and coefficient of inbreeding (*Fis*)

If any of the above-listed information were not reported in the paper, this was recorded as “Not-available.”

Methods and data-reporting customs varied, with many papers providing only the approximate location of the sampled populations described in text or indicated on maps. In several cases, the reported coordinates did not match with the name of the sampling area or were not available within the articles (Thiebaut et al. 1982; Belletti et al. 1996; Chybicki et al. 2009; Comps et al. 2001; Comps et al. 1990; Demesure et al. 1996; Dounavi et al. 2010; Jump et al. 2006; Nowakowska and Sułkowska 2011; Sander et al. 2000; Sulkowska 2010; Wang 2003, 2004); for these publications, approximate coordinates and country were extracted from the location name, the field site, or the maps using QGIS software (https://www.qgis.org/en/site/). Genetic indices were also reported in a variable manner, and in 8 publications, genetic indices were averaged across all studied populations (Table S1).

### Generation of additional nuclear microsatellite data

In addition, a previously unpublished nuclear microsatellite dataset was included, because of its wide and even geographic coverage of the distribution range (see description of sampling in Ulaszewski et al. 2021). Briefly, trees from two provenance trials, situated in Siemianice (Barzdajn and Rzeznik 2002) and in Choczewo (Chmura and Rozkowski 2002), were sampled with 10–20 individuals per seed source population, representing a total of 85 different populations across Europe (see Dryad dataset for this paper). DNA was extracted using the GeneMATRIX Plant & Fungi DNA Purification Kit (EURx, Poland), and a selection of 20 microsatellite markers was used for genotyping: csolfagus_29, csolfagus_31, csolfagus_19, csolfagus_05, csolfagus_06, DE576-A-1, DUKCT-A-1, DZ447-A-1, EJV8T-A-1, EMILY-A-1, ERHBI-A-1, Fc3, Fc6, EEU75-A-1 (Lefevre et al. 2012), CsCAT15 (Marinoni et al. 2003), Fc5, Fc9 (Ueno et al. 2009), FS1-15 (Pastorelli et al. 2003), sfc0036, and sfc1143 (Asuka et al. 2004). Obtained PCR products were sized using a capillary sequencer ABI PRISM 3130XL® (Applied Biosystems, Foster City, CA, USA), and allele calling was performed using GENESCAN 3.7 and GENOTYPER 3.7 software (Applied Biosystems, Foster City, CA, USA). Allele binning was done manually after plotting of fragment size distributions for each locus (Guichoux et al. 2011).

### Calculation of genetic diversity indices

For studies using nuclear microsatellites, primer sequences (listed in Table S4) were aligned on BLAST (https://blast.ncbi.nlm.nih.gov/Blast.cgi) to prevent redundancy (i.e., treating primers with the same sequence but different names as different loci). When *He* and genotypic data were not available, *He* was calculated from the allelic frequencies, if provided (B.Thiebaut 1982; Gömöry et al. 1998; Dounavi et al. 2010). As populations, we considered groups of *F. sylvatica* for which distinct diversity indices were reported. If populations were not clearly defined or were grouped in large zones, the average number of populations per country or zone was considered, as indicated in the publication. For studies which used more than one marker system (Bilela et al. 2012; Cuervo-Alarcon et al. 2018; Müller et al. 2018; Paffetti et al. 2012), genetic diversity indices were extracted for both molecular marker types, and the correlation between genetic diversity indices was estimated (Spearman’s rank test). Distributions of compiled reported values of genetic diversity obtained with diverse molecular markers were compared using a Kruskal–Wallis test, and after verifying that these differed by marker (Kruskal–Wallis’s test = 986.45, *p* < 2.2e-16), analyses were conducted separately for each marker system. Genetic diversity from SNP data, reported as *He* or as nucleotide diversity, was analyzed as described in the Supplementary Material. Since nuclear microsatellites were the most frequently used marker types among the considered studies (Table S1), we focused further analysis on this marker type. Mean genetic diversity per locus was recorded from the studies or computed from allelic frequencies, where reported.

### Construction of the meta-dataset

Among the 24 studies based on nuclear microsatellites, 11 original genotype datasets were retrieved and compiled in a meta-dataset together with the dataset from the present study (Table 1, Supplementary Information), while for the rest of the studies, we only calculated mean and standard deviation of reported genetic diversity metrics (see Table S5). Allelic frequencies and genetic diversity statistics were re-computed using the *adegenet* R package on each dataset singularly and afterwards combined in a unique table. The following analyses were based on expected heterozygosity (*He*). Studies were grouped according to the set of microsatellite loci used for genotyping, resulting in five clusters of studies using a similar set of loci within each cluster (Fig. S2). To test whether the array of loci chosen by the authors influenced the calculated *He*, a Kruskal–Wallis test and subsequent Dunn’s post-hoc test with the Bonferroni correction were performed on *He* values from these different clusters. Since *He* is derived from loci with alleles which differ in number and frequencies and therefore polymorphism, the metrics cannot be compared across studies. In other words, the same population genotyped with highly polymorphic loci would present a higher *He* than if it were genotyped with less polymorphic loci. This makes values of *He* computed by studies using different genotyping kits incomparable and a method for reducing the difference across-studies desirable.

**Table 1:** Original datasets of nuclear microsatellite genotypes used for the scaling approach and the interpolation of genetic diversity across the range of *Fagus sylvatica*

Article	Country	Number of loci	Number of populations	Sample size
Lander et al. 2011	France	13	51	1932^a^
Lefevre et al. 2012	France	16	4	40–45
Piotti et al. 2012	Austria and France	4	4	376–427
De Lafontaine et al. 2013	France and Spain	16	65	40
Gauzere et al. 2012	France	13	3	137–194
Rajendra et al. 2014	Germany	9	3	10
Sjolund et al. 2017	Germany, France, Italy	11	6	100–170
Cvrkova et al. 2017	Czech Republic	12	13	390^a^
Oddou-Muratorio et al. 2018	France	13	3	722^a^
Müller et al. 2018	Germany	9	22	150^a^
Cuervo-Alarcon et al. 2018	Switzerland	13–76	12	25
Ulaszewski et al. 2021	Nine European countries (distribution, Fig. 4 and Dryad dataset)	20	85	10–20

^a^Total number of sampled trees

### The min–max scaling approach and its validation

Reddy and Rosenberg (2012) derived a formula for the theoretical maximum of homozygosity (*H*), valid for a variable polymorphic genetic locus treated as indeterminate, with at most *K* alleles. Because *H* and *He* sum to one, we used this formula to standardize the *He* values (*He scaled*) across studies. We retained data from loci with unusually low levels of polymorphism which were estimated by comparing the mean *He* across the loci with a Kruskal–Wallis test and post-hoc test, to avoid further loss of genetic information. Using this method, the differences in scaled *He* are expected to reflect the population size and its historical changes. According to Theorem 2 from Reddy and Rosenberg (2012), the theoretical range of *H* given a fixed maximum number of alleles *K* ≥ *2* present in a population is as follows:

$$\frac{\mathrm{KM}^2-2\mathrm M+1}{\mathrm K-1}\leq\mathrm H\leq1-\mathrm M\left(\lceil\mathrm M^{-1}\rceil-1\right)\left(2-\lceil\mathrm M^{-1}\rceil\mathrm M\right)$$

where *M* is the frequency of the most frequent allele for each locus. These values were used to scale the *H* for each locus *i* and each population *j* according to the following formula:

$$\mathrm H{\;\mathrm{scaled}}_{\mathrm i,\mathrm j}=\frac{{\mathrm H}_{\mathrm i,\mathrm j}-\min\left(\mathrm{theoretical}\;{\mathrm H}_{\mathrm i,\mathrm j}\right)}{\max\left(\mathrm{theoretical}{\;\mathrm H}_{\mathrm i,\mathrm j}\right)-\min\left(\mathrm{theoretical}\;{\mathrm H}_{\mathrm i,\mathrm j}\right)}$$

where min(theoretical *H*_*i,j*_*)* and max(theoretical *H*_*i,j*_*)* are the minimum and maximum theoretical values of *H* for each locus *i* and each population *j*. Scaled *He* was subsequently calculated as *1–H* and averaged across loci. We conducted a Kruskal–Wallis test between the *He* values from different studies before and after scaling, in order to check whether scaling reduced study-to-study differences as expected.

We sought to understand whether the certainty in assessing geographic patterns could be reduced by potential differences in polymorphism between loci (locus effect) and by varying polymorphism of the same locus across the species range (discovery effect). Furthermore, authors sampling in regions with high diversity may preferably have used one set of loci, while authors in regions with low diversity may have tended to use a different set. Therefore, *He* may significantly depend on both locus identity and geographic location. In order to test the utility of the scaling approach for correcting these types of bias, we used the novel, range-wide nuclear microsatellite dataset described above and in Ulaszewski et al. 2021, to perform two re-sampling studies.

First, we tested whether the use of loci developed in different parts of the range (discovery effect) could lead to ascertainment bias. The pool of loci used by Ulaszewski et al. could be split into three groups of kits developed independently in different studies, as shown in Fig. S4. Mean *He* was computed considering data from these three kits separately and scaled with our approach (for details, see the references indicated in Fig. S4). Afterward, raw and scaled *He* values from the different kits were correlated with latitude, longitude, and distance from origin.

Second, the Ulaszewski dataset was split geographically in order to simulate a geographic bias in locus polymorphism. Specifically, we took a subset of the Ulaszewski dataset such that either the eastern populations had more polymorphic loci only and the western less polymorphic loci only or so that the southern populations had more polymorphic loci only and the northern had less polymorphic loci only. As a control, we also split the populations and the loci randomly. We then tested correlations of these artificial, biased datasets against latitude, longitude, and distance from hypothesized origin, using either the raw or scaled *He* values, and compared the results (Fig. 5, Figs. S5 and S6).

Furthermore, we tested whether the correlation of raw *He* from the combined meta-dataset against latitude, longitude, and distance from hypothesized origin supported the same conclusions as did the correlation against the scaled *He*.

### Range-wide patterns of genetic diversity

To compare patterns of genetic diversity derived from raw and scaled *He* across the species range, we correlated these against latitude, longitude, and distance from hypothesized origin. We chose latitude because a northward expansion from southern glacial refugia should result in decreasing with latitude (from south to north in Europe). We chose longitude because a westward expansion from an eastern origin should result in a positive correlation of genetic diversity with longitude (from west to east in Europe). For the hypothesized origin, we chose Slovenia and the eastern Alps, locations which were proposed by Magri (2008) and Magri et al. (2006) to be the main source areas for the colonization of central and northern Europe by beech. All patterns may be interrupted by the presence of biogeographical barriers and habitat discontinuity. Additionally, Magri and colleagues (2006) also suggested refugia in southern France and the northern Iberian Peninsula, which have also been identified as separate gene pools by Postolache et al. 2021; however, there is no genetic evidence that these lineages expanded after the LGM. *He* of these populations is supposed not to correlate with geographic distance from the Slovenian refugia: these regions are highlighted in Fig. 4 as blue and orange points, respectively.

For visualization, scaled *He* values were shown both as data points and also interpolated over the species range. Ordinary block kriging (2 × 2) was computed with the R function *idw* (*gstat* package) using a power parameter of 5. The map was subsequently masked to the distribution range of beech in Europe provided by EUFORGEN (http://www.euforgen.org). A leave-one-out cross-validation approach was subsequently used to check the effectiveness of kriging parameters and to construct confidence intervals on the estimates (Refaeilzadeh et al. 2009), by removing one observation at a time from the original dataset and repeatedly estimating a pseudo-value corresponding to each omitted point according to the jackknife procedure.

## Results

### Overview of studies

**Fig. 2:**
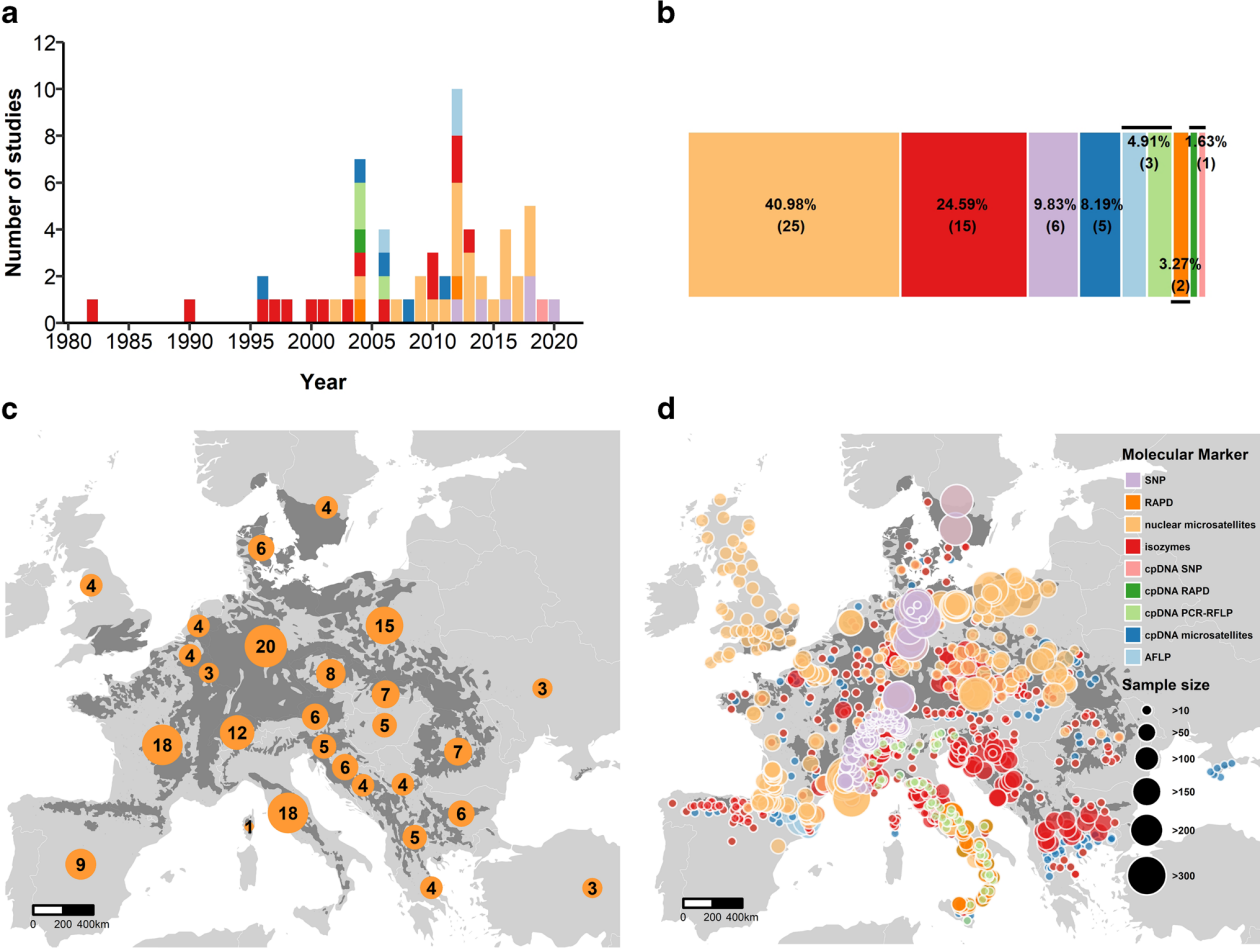
Geographic distribution of the studies of *F. sylvatica* genetic diversity included in this review. **a** Number of studies across the years coded by marker type; legend applies to **a**, **b**, and **d**. **b** Percentages and number of studies using each molecular marker system. **c** Number of studies per country. **d** Sample size and molecular marker for each analyzed population of *F. sylvatica*

Studies measuring the genetic diversity of *F. sylvatica* began to be published in the 1980s, and numbers increased steadily over the last 20 years (Table 1, Fig. 2a, and Supplementary results summarizing raw results on genetic diversity and structure from the systematic review). We were able to obtain data from 1044 populations across 27 countries (Fig. 2c). Genetic data were heterogeneously distributed across the geographic bioclimatic niche of *F. sylvatica*, being richest in the center-south of the range, including Switzerland, Germany, Czech Republic, Poland, southern France, and Italy, and less dense in the north and east (Fig. 2d). DNA was most often extracted from leaves (25) and leaf buds (21) or both. Sample size, age profiles of sampled trees, sampling year and season, sampling area, and inter-tree distance were not consistently reported (Table S1).

### Overview of marker systems

Reviewed publications were based on nine molecular marker systems (Fig. 2b, Table S5). Isozyme-based analyses followed the methodology described by B.Thiebaut et al. 1982, Merzeau et al. 1989, and Müller-Starck 1996, for a total of 16 enzymes used (Table S3). The nuclear microsatellite analyses were based on the PCR multiplex kits designed by several authors, for a total of 63 loci (Table S4). The pairs of primers used for cpDNA microsatellite analyses were described by Demesure et al. (1996), Taberlet et al. (1991), and Weising and Gardner (1999), while AFLP analyses mostly followed a modified version of the original protocol published by Vos et al. (1995). Finally, RAPD analyses were performed following the PCR conditions reported by Emiliani et al. (2004). Differently named primers used in different publications did not overlap in BLAST alignments and thus can be considered to represent different loci.

The majority of studies investigated nuclear markers. For a comprehensive and more detailed analysis of chloroplast genetics across the range of *F. sylvatica*, we refer to the studies conducted by Magri (2008), Magri et al. (2006), Vettori et al. (2004), and for Greece by Hatziskakis et al. (2009). Notably, the complete chloroplast genome sequence of *F. sylvatica* has recently been published (Mader et al. 2019), and novel chloroplast SNPs were detected using reduced representation library sequencing methods on geographically widely distributed individuals (Meger et al. 2019).

### Comparison of expected heterozygosity based on nuclear microsatellites

In this study, we present scaled *He* for *F. sylvatica* populations from a metadataset comprising 12 individual datasets using nuclear microsatellite markers. Allelic size ranges were broadly consistent across studies using the same loci (Fig. S1). Nevertheless, for several loci, the alleles recorded were different only for one repeat, showing an alternate fashion across publications, which could suggest inconsistencies in PCR fragment binning and allele naming across laboratories, rather than different allele identities. Moreover, the genotyping kits used in the 26 publications varied in terms of the number and identity of loci (Fig. S2), hampering the comparison of results from different publications. As expected, *He* values differed significantly among clusters of studies defined by different sets of loci (Kruskal–Wallis’s test, *He* by group, *H*(5) = 297.65,* p* < 2.2e-16). In this comparison, the difference in polymorphism among investigated loci cannot be separated from differences in the population size and in the local demographic history. Therefore, we proceeded with a scaling approach to ameliorate these study-, population-, and location-specific differences.

### Range-wide patterns of expected heterozygosity and validation of the min–max scaling approach

**Fig. 3:**
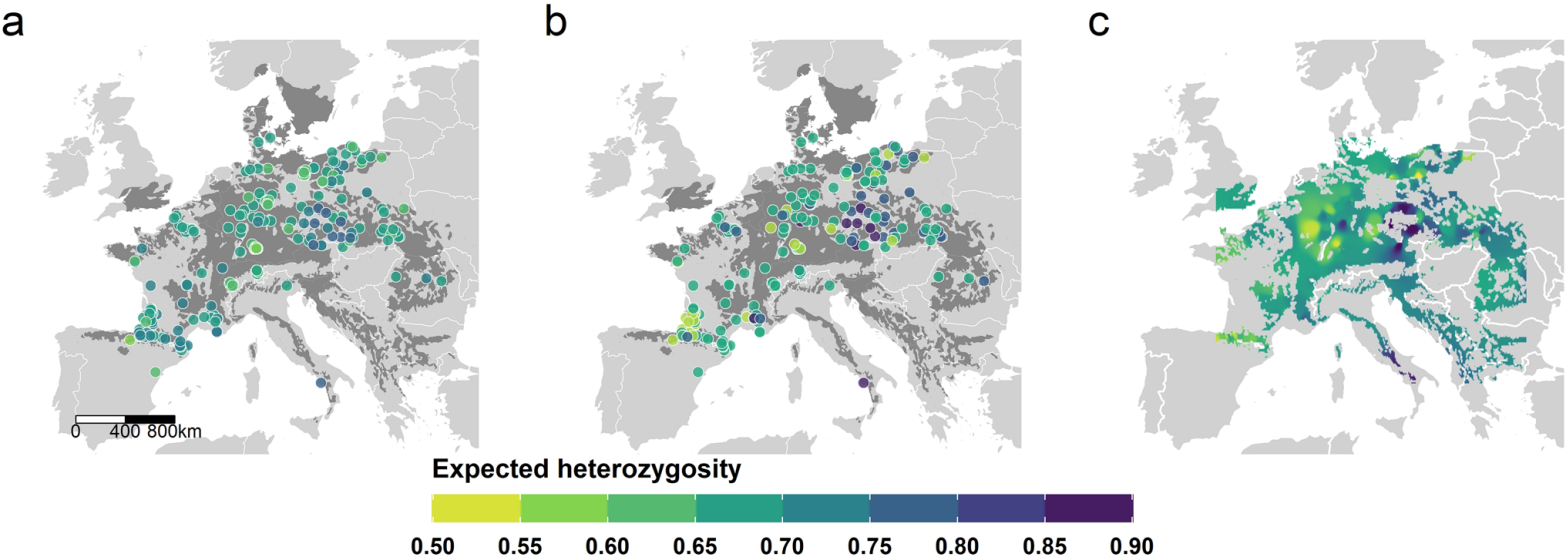
Spatial distribution of *He* across 227 *F. sylvatica* populations from nuclear microsatellite markers. Raw *He* retrieved from the studies (**a**), scaled *He* (**b**), and interpolation of scaled *He* (**c**, interpolation distance weighting, power = 5) across the distribution range of *F. sylvatica*. Legend and spatial scalebar annotation apply to all maps

**Fig. 4:**
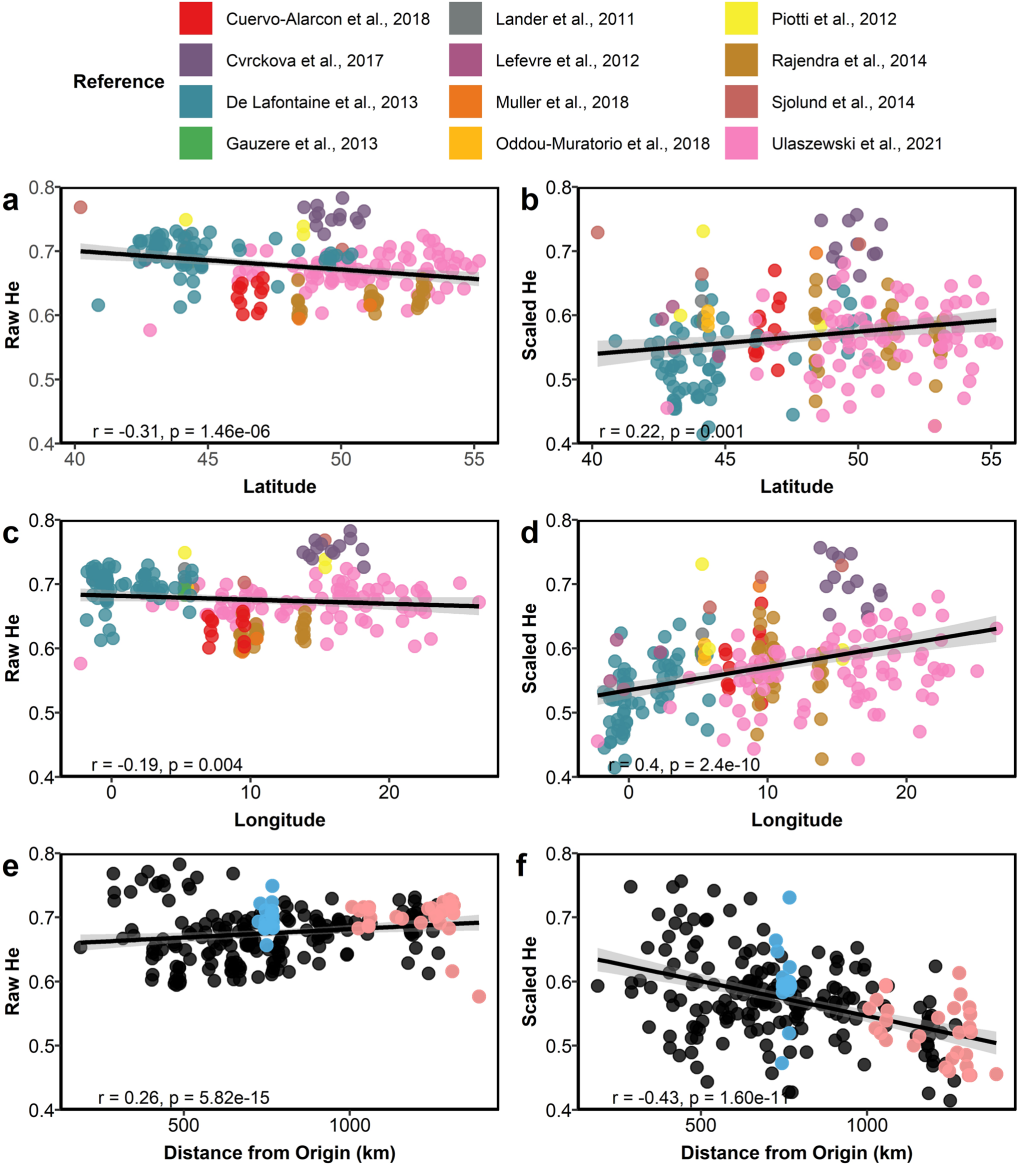
Correlation between non-scaled (**a**, **c**, and **e**) or scaled (**b**, **d**, and **f**) genetic diversity (*He*) from nuclear microsatellite markers; geographic variables, latitude (**a** and **b**) and longitude (**c** and **d**) with points color-coded by study; and distance from a putative origin area (Magri et al., 2006) corresponding to the Slovenian region. Blue and orange points denote populations in the French Alps and in the Iberian Peninsula, respectively, which are proposed to be additional refugia for genetic diversity (**e** and **f**)

Our min–max scaling approach resulted in a distribution of scaled *He* values between 0.41 and 0.76, with an average of 0.57 ± 0.067 (mean ± SD, Fig. 3 and Fig. S3). The root-mean of squared residuals (RMSE) for the leave-one-out cross-validation of the interpolated distribution was 0.0648 km, and confidence intervals computed to verify interpolation accuracy ranged between 0 and 0.4 (Figs. S7 and S8). The highest scaled value was found north-east of the Alps (Czech Republic, southern Poland, Austria) and southern Italy. Scaled *He* revealed a decreasing trend in diversity from east to west using the whole meta-dataset (*r*_*s*_ = 0.4, *p* = 2.4e-10, Fig. 4d), while a weak but opposite trend was observed with the unscaled data (*r*_*s*_ =  − 0.19, *p* = 0.004, Fig. 4c). Similarly, scaled *He* increased at higher latitudes (*r*_*s*_ = 0.22 *p* = 0.001, Fig. 4b), while raw *He* decreased as the latitude increased (*r*_*s*_ =  − 0.31, *p* = 1.46e-06, Fig. 4a). The correlation between scaled *He* and geographic distance from the refugia suggested by Magri (2008) and Magri et al. (2006) was negative and statistically significant (*r*_*s*_ =  − 0.43, *p* = 1.6e-11, Fig. 4f); in contrast, the raw *He* values showed a positive and significant increase in diversity with the increase of the distance from the refugia (*r*_*s*_ = 0.26, *p* = 5.8e-15, Fig. 4e). In sum, scaled *He* values decreased with distance from refugia and hypothesized origins, while raw *He* showed the opposite trend.

**Fig. 5:**
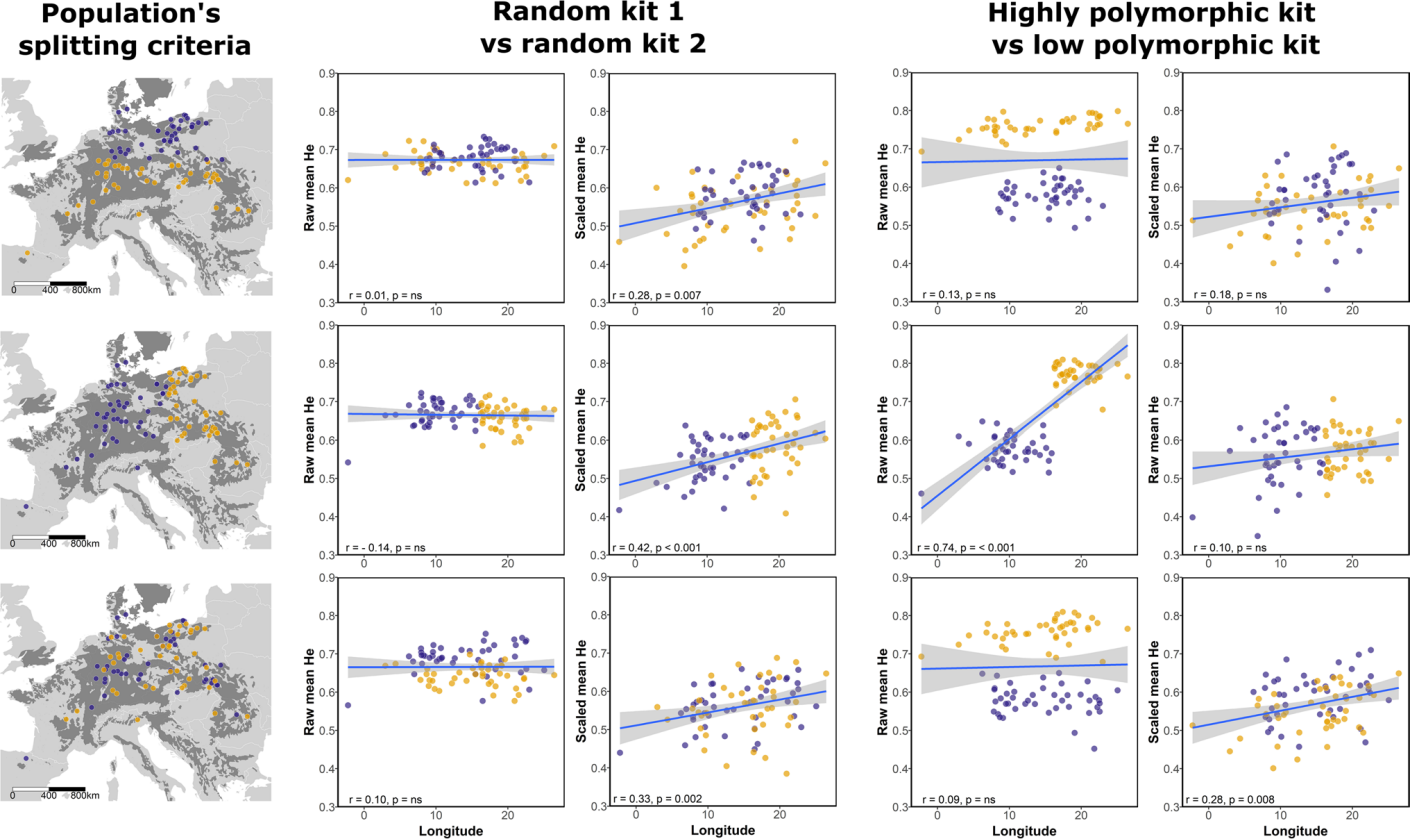
Correlation scatter plots (linear regression as blue line with confidence interval as gray area) between longitude and the raw and scaled *He* dataset from Ulaszewski et al., 2021. Populations were splitted into north (blue) versus south group (yellow), west (blue) versus east (yellow) group, and two random groups. Loci were divided either randomly or according to the polymorphism level, lower (blue) or higher (yellow). Spearman’s *R*_*s*_ and *p*-values are reported inside the correlation plot

The effect of scaling was also evident after splitting the dataset into different subsets by geographic origin and by locus (systematic or random, see Table 2, Fig. 5, and Figs. S5 and S6). Generally, data which had been artificially clustered to test bias became more homogeneously distributed after scaling. Regression coefficients of the relationships between longitude and raw mean *He* were either near zero and not statistically significant or close to 1 and strongly significant due to the polymorphism bias (Table 2 and Fig. 5). In contrast, regression coefficients for the scaled data were consistently positive with an *r* between 0.1 and 0.42 and were statistically significant when loci had been split randomly (i.e., random kit assignment or random geographic assignment). Again, scaling was effective in eliminating spurious significant correlations and reducing the geographic bias for the relationships between *He* and latitude and distance from origin (Figs. S5 and S6).

**Table 2:** Summarized *r*_*s*_ and *p* values for Spearman’s correlation tests between the raw and scaled dataset from Ulaszewski et al., 2021 and latitude, longitude and distance from origin. See also Figs. 5, S4, S5, and S6

Scenario	Correlation	Raw mean *He*		Scaled mean *He*	
		*r*_*s*_	*p*	*r*_*s*_	*p*
Complete dataset	Latitude	0.173	0.113	0.119	0.277
	Longitude	0.157	0.1513	0.324	0.00216*
	Distance from origin	0.080	0.4631	0.016	0.894
North–South Loci randomly distributed	Latitude	0.308	0.0040*	0.180	0.099
	Longitude	0.008	0.939	0.287	0.007*
	Distance from origin	0.237	0.0223*	0.091	0.406
North less polymorphic South more polymorphic	Latitude	− 0.713	1.73e-14*	0.259	0.0167*
	Longitude	0.129	0.238	0.185	0.088
	Distance from origin	− 0.548	5.6e-08*	0.130	0.232
West–East Loci randomly distributed	Latitude	0.128	0.242	0.111	0.309
	Longitude	− 0.142	0.193	0.421	5.95e-05*
	Distance from origin	0.27	0.805	0.039	0.724
West less polymorphic East more polymorphic	Latitude	0.107	0.33	0.081	0.462
	Longitude	0.736	9.60e-16*	0.101	0.358
	Distance from origin	0.123	0.264	0.012	0.912
Random groups Loci randomly distributed	Latitude	0.103	0.345	0.117	0.287
	Longitude	− 0.071	0.514	0.327	0.002*
	Distance from origin	− 0.0037	0.973	0.103	0.347
Random groups Less polymorphic More polymorphic	Latitude	0.108	0.324	0.095	0.385
	Longitude	0.087	0.427	0.285	0.008*
	Distance from origin	0.117	0.285	0.054	0.621

Furthermore, we observed that raw *He* values tend to cluster together according to the study of origin, suggesting that the specific loci used have a strong influence on *He*. For instance, De Lafontaine et al., 2013, used 16 highly polymorphic loci and report relatively high *He* values (Figs. S1 and S2). Instead, the scaled *He* values have a similar spread among studies. The Kruskal–Wallis test between the *He* values from similar genotyping kits (Fig. S2) showed statistically significant differences for both raw and scaled *He*; nevertheless, this difference is reduced for the scaled data (raw *He* x group of studies, Kruskal–Wallis’s test, H(3) = 121.42, *p* < 2.2e-16, scaled *He* x group of studies, Kruskal–Wallis’s test H(3) = 58.155, *p* = 1.457e-12). This finding indicates that the scaling removed most of the locus-specific effects related to the number of alleles, but did not completely eliminate differences among studies. Scaling was effective in reducing potential ascertainment bias, which we simulated by splitting the largest nuclear microsatellite dataset reported here, from Ulaszewski, using two criteria. When mean *He* was computed only from a subset of loci belonging to specific genotyping kits (Fig. S4), scaling the data resolved the likely spurious, statistically significant correlation between raw mean *He* from kit 2 and distance from origin, while all other correlations with latitude and distance from origin were very weak (*r* < 0.1) or positive and not statistically significant. In the case of longitude, the scaling method strengthened the positive correlation to mean *He* computed from each kit, resulting in Spearman’s correlation coefficients between 0.213 and 0.328, with all *p*-values < 0.5. In all cases, the scaled mean *He* values were smaller on average than the raw mean *He* values.

Correlations of *He* from the large single dataset reported here against latitude, longitude, and distance from the refugia proposed by Magri were not statistically significant, except for the case of scaled *He* values against longitude (Spearman’s correlation, *r*_*s*_ = 0.33, *p* = 0.002).

## Discussion

We present a systematic review of studies on *Fagus sylvatica* genetic diversity, based on 50 publications spanning nearly 4 decades, and one additional study introduced here, as a reference for population genetic studies in European beech. The field of molecular genetics has dramatically changed during this time, and it is challenging to combine these studies in order to improve general knowledge about the spatial distribution of genetic diversity across the species range. Genetic diversity metrics generally vary as a function of marker type, genomic location, and numbers of analyzed DNA sequences. Using a larger number of loci from different original studies is a good first step toward the estimate of genome-wide diversity allowed by approaches using thousands of SNPs and other genomic markers. The geographic coverage of SNP-based studies in *F. sylvatica* is still patchy as a proportion of the total range, concentrated in the French Alps and Switzerland (Fig. 2). Many more SNP datasets are expected to be generated in the future, and approaches are in continuous development. Thus, we feel it is premature to review that topic, and simply note here that recent SNP studies in beech focus on signatures of adaptation to abiotic factors (Capblancq et al. 2020b; Cuervo-Alarcon et al. 2021; Oddou-Muratorio et al. 2021; Pfenninger et al. 2021; Meger, Ulaszewski and Burczyk 2021; Postolache et al. 2021).

Although SNPs, like other marker types, do not permit unbiased interpretation, they do have advantages over earlier markers conferred by their deeper and broader sampling of whole genomes, one reason they are emerging as preferred (Fig. 2). Yet, despite the increasing affordability of the sequencing step, the overall process of generating and analyzing genomic markers is still lengthy and expensive, and sampling distribution, breadth, and depth remain important optimization parameters. Here, we describe a way for researchers to combine and interpolate from existing data in order to synthesize existing knowledge of species genetic diversity and guide sampling designs in future genomic studies.

We focused on the to-date most frequently used marker type, nuclear microsatellites. We developed a novel approach based on population genetics theory to scale *He* computed from different sets of microsatellite loci, allowing the combination of multiple individual studies to discern broader trends, which could be relevant beyond the scope and subject of this systematic review. This procedure enabled the detection of plausible geographic gradients in *He* of *F. sylvatica* populations derived from nuclear microsatellites (Fig. 4 b, d, f) which could not be observed from raw data affected by locus-, location-, and study-specific biases (Fig. 4 a, c, e). Our validation of the scaling approach indicates that it is able to substantially ameliorate artifacts due to polymorphism differences among (Figs. S1 and S3) and within loci and is useful not only for combining studies, but for reducing discovery effects (the tendency for individual loci to vary in their inherent polymorphism across a species range) in the study presented here, which has unusually broad and even geographic coverage. The scaled scores still vary by locus, but reveal more variation at each locus, i.e., they are less clustered, and the mean of the scaled values tends more toward the central tendency of the whole meta-dataset. We performed validation tests by splitting the large dataset reported here in different ways to simulate bias and found that in all cases, the introduced bias was ameliorated by scaling (Fig. 5 and Figs. S4, S5, S6). Thus, we suggest that the scaled *He* values allow better assessment of relative *He* values within and across loci. The comparison of raw with scaled data indicates that *He* at low longitude and latitude is systematically overestimated in the raw data (Fig. 4). This corresponds to a choice of microsatellite markers at low longitudes and latitudes for which the scaled *He* is almost always lower than the raw *He* (Figs. S1, S2, S3). Interestingly, scaling has relatively less effect on the study from Cvrčková and colleagues (2017), which is unique in using markers from across all three sets of microsatellites commonly used for *F. sylvatica* and selecting among these loci which tend to have higher polymorphism (Figs S1 and S2). The geographic patterns which emerge from the scaled *He* data (Figs. 3, 4), indicate a loss of genetic diversity in *F. sylvatica* populations with distance from the species origin and its refugia.

The interpolated scaled *He* patterns we describe here from nuclear microsatellites are consistent with higher genetic variation in beech stands located in the north of the Alps, in particular corresponding to the Czech and Slovakian populations (Fig. 3). This area, proposed by Comps et al. (2001) and Magri et al. (2006) to be colonized 8000 ya from Slovenia, could be considered a hotspot of genetic diversity, as different source populations coming from refugia in Slovenia and Moravia may have merged in this area, with consequent exchange among the diverse gene pools. Nevertheless, given that the Czech populations with highest *He* were collectively analyzed in the same study (Cvrčková et al. 2017), the possibility that differences in experimental design influence this result cannot be ruled out, even in our filtered and scaled meta-dataset. Our findings are similar to those from Oddou-Muratorio et al. (2021) who reported above-average values of genetic diversity in northern and south-western populations (southern France and northern Spain); however, they observed lower *He* in southeastern Europe (Italy, Greece, and the area between Poland and Ukraine). Very few or no data were available for these southeastern populations, and thus, our interpolated map is expected to be less accurate in those areas. In line with other markers, southern French and Italian regions display relatively high nuclear microsatellite-derived *He*, with less dense nuclear microsatellite data in Italy. The trends revealed by these combined, filtered and scaled microsatellite datasets were not clearly detectable when considering only one single dataset which incorporates the largest geographic variation in *F. sylvatica* yet published in a single study (Ulaszewski and Burczyk reported here), except for the strongest correlation (longitude) (Fig. 4, Table 2). These trends are therefore strengthened by the addition of filtered and scaled data from other studies, which provide a geographically more complete picture of *F. sylvatica*’s genetic variation.

The spatial distribution of genetic diversity supports the hypothesis that *F. sylvatica* colonized its current habitats from the south-west of the Balkan Peninsula, Slovenia and north Dalmatia, and from a central European refugium, southern Moravia and southern Bohemia (Magri et al. 2006; Kempf and Konnert 2016; Cuervo-Alarcon et al. 2018; Capblancq et al. 2020b). Unfortunately, we did not identify any studies from the south-west of the Balkan Peninsula. In contrast, in agreement with the above hypothesis, we found the highest levels of heterozygosity in populations from the Czech Republic and from southern Poland, which might represent descendants of the central European refugium. However, high levels of genetic diversity may also result from admixing of different genetic lineages from different colonization routes merging in this area (Oddou-Muratorio et al. 2021). Slovenian beech populations in the eastern Alps, for which nuclear microsatellite-based data were not available, could have migrated toward the north and admixed with one or more potential refugia located in the Moravian region as proposed by Magri et al. (2006), which migrated to the west (Postolache et al. 2021). Our scaled data also indicated unusually high levels of genetic diversity in some populations from southern France, another proposed refugium, although the pattern is not general: many nearby populations have relatively low genetic diversity in our analysis.

Our range-wide scaled data can be contrasted with another recent range-wide study which, however, used SNPs from candidate genes—primarily genes annotated as being involved in phenology and stress response (Postolache et al. 2021) (Postolache et al. 2021). The authors found that *F. sylvatica* populations are separated into three main clusters in southeastern, southwestern, and northern Europe by biogeographical barriers corresponding to the English Channel, the Baltic Sea and plains in western Germany and north-western France, the Alps, and the Carpathians, along estimated effective migration surfaces (EEMS). Our scaled genetic diversity derived from nuclear microsatellites is not in accordance with the genetic diversity map proposed by this study, where the highest genetic diversity is in French populations, while the area of Czech Republic and Southern Poland show a lower-then-average *He*. This may indicate differences between patterns of polymorphism for genes involved in adaptation to diverse environmental conditions across the range of European beech, versus neutral genetic structure revealed by microsatellites. We also note that both SNP sets used by Postolache et al. (2021) were based on samples from France (Ventoux for Lalague et al. 2014 and “Les Gillet” for Lesur et al. 2015), which can cause some ascertainment bias. Interestingly, both our analysis and that of Postolache and colleagues indicate higher relative genetic diversity in the region of southwest Poland and northern Slovakia. High genetic diversity can indicate a zone of admixture of different lineages, but also a refugium, and our analysis cannot distinguish between the two.

The results presented here are consistent with the hypothesis that mountain barriers have shaped the diversity of local microrefugia. These stands, isolated from the main genetic pool, are suggested to be located in the western Pyrenees and south-eastern France (De Lafontaine et al. 2013), in the Apennines and in the Balkan peninsula (Magri 2008), regions where we have identified populations with relatively high genetic variation. Apart from these supposed microrefugia, analyses based on different nuclear and chloroplast markers converge toward a homogeneous genetic structure of *F. sylvatica* across central, eastern, and northern Europe, which may suggest that this species is able to adapt to a variety of environmental stresses and survive in diverse environments. Overall, various markers indicate higher genetic diversity for *F. sylvatica* in the north-east than in the western part of the species distribution.

### Limitations and outlook

We focused on neutral genetic diversity, which is an important indicator to understand the demographic history of a species. Neutral diversity should be considered in combination with functional genetic diversity in order to infer the ability of populations to cope with environmental change (Yıldırım et al. 2018). The majority of genetic diversity studies in *F. sylvatica* use neutral markers, although this is changing with the advent of genomic methods. While we attempted an exhaustive review of the peer-reviewed literature, we did not include preprints, which are expected to preferentially use genome-wide approaches. Here, we note three studies discussed in the text of our review which were published too late for inclusion in our main analysis, reporting new microsatellites from refugia in southeastern France (Lander et al. 2021) and SNP datasets from Germany (Pfenninger et al. 2021) as well as from 64 populations across the species range (Postolache et al. 2021).

In several cases, the results of the studies reviewed here are accessible in databases and therefore available for building an integrated dataset for scientific analysis and comparison, but in many cases, the data or metadata of the study could not be fully retrieved either from the publication or from a database. Sampling coordinates, sample size, and genetic parameters were occasionally not reported on a per-population basis. Overall, sampling strategies were highly variable in terms of spatial distribution and numbers of sampled individuals, numbers, and types of genetic markers used. The resulting picture of genetic variation is heterogeneous and fragmented. It has been shown by Landguth et al. (2012) that to achieve sufficient statistical power, amplifying more and more variable loci is likely more effective than increasing the number of individuals sampled. In addition, we observed inconsistencies when reporting the names of the loci used for the genotyping and likely also of the alleles.

The scaling approach developed here for nuclear microsatellite data also has limitations. Our proposed scaling is based on Theorem 2 developed by Rosenberg and Jakobsson (2008) and thus implies assumptions on the values of *H*, *K*, and *M* which are valid under the condition of Hardy–Weinberg’s proportions (Weir 1996). The number of alleles is fixed as *K* ≥ 2, and *M*, the frequency of the most frequent allele, is given as ∈ (1/K, 1). Upper and lower bounds of *H*, the homozygosity, are functions of *M* and vice versa, and their range is dependent on *K* (Rosenberg and Jakobsson 2008). Thus, this approach likely does not eliminate all undesirable differences among loci or among studies, consistent with our observations and validation tests. We chose min–max scaling as it constrains values between 0 and 1 based on a scale (difference of minimum and maximum value) which is easy to interpret and without incorporating other assumptions about data distribution.

We used the scaled *He* values to investigate potential patterns in genetic structure using simple correlations with latitude and longitude, which provide little information about the historical and environmental drivers of observed relationships. Remote sensing and earth observation technologies constitute spatially resolved and contiguous approaches increasingly promising in linking genetic data to environmental information relevant to conservation of plant genetic resources, as is now established for the analysis of many plant traits (e.g., Asner et al. 2017; Wang et al. 2020). Additionally, models and simulation studies projecting possible future distributions and challenges for *F. sylvatica* under probable climatic scenarios are in continuous development (e.g., Capblancq et al. 2020a, b; Falk and Hempelmann 2013; Kramer et al. 2010). The combination of these different types of information and approaches with previous knowledge may help to prioritize populations for conservation or intervention, mitigating the impact of contemporary environmental changes on natural populations.

## Supplementary Information

Below is the link to the electronic supplementary material.

Supplementary file1 (JPG 511 KB)

Supplementary file2 (JPG 454 KB)

Supplementary file3 (JPG 486 KB)

Supplementary file4 (JPG 343 KB)

Supplementary file5 (JPG 3126 KB)

Supplementary file6 (JPG 1651 KB)

Supplementary file7 (PNG 7402 KB)

Supplementary file8 (PNG 4572 KB)

Supplementary file9 (PNG 4371 KB)

Supplementary file10 (JPG 336 KB)

Supplementary file11 (JPG 147 KB)

Supplementary file12 (DOCX 114 KB)

## Data Availability

Data archiving statement: Datasets which were not already publicly available at the time of submission are provided in the supplementary material.

## Code availability

Statistical analyses were conducted using R version 3.6.3 (R Core Team 2020) and RStudio version 1.1.463 (Rstudio Team 2019), packages tidyverse (Wickham and Henry 2019), dplyr (Wickham et al. 2019), tidyr (Wickham and Henry 2019), rstatix (Kassambara 2020a), dunn.test (Dinno 2017), EnvStats (Millard 2014), geosphere (Hijmans 2019), adegenet (Jombart 2008), pegas (Paradis 2010), and gstat (Pebesma 2004), and SNP data were analyzed in VCFtools version 0.1.16 (Danecek et al., 2011). Figures were generated using ggplot2 (Wickham 2016), ggpubr (Kassambara 2020b), sf (Pebesma 2018), raster (Hijmans 2020), ggspatial (Dunnington 2020), ggridges (Wilke 2020), pheatmap (Kolde 2019), RColorBrewer (Neuwirth 2014), cowplot (Wilke 2016), tmap (Tennekes 2018), and viridis (Garnier 2018) or base R plotting arguments. Scripts are available in the supplementary material.

## References

[CR1] Aerts R, Jonnay O (2011). Forest restoration, biodiversity and ecosystem functioning. Plant Ecol.

[CR2] Aranda I, Cano FJ, Gasco A, Cochard H, Nardini A, Mancha JA, Lopez R, Sanchez-Gomez D (2015) Variation in photosynthetic performance and hydraulic architecture across European beech (Fagus sylvatica L.) populations supports the case for local adaptation to water stress. Tree Phys 35:34–46 10.1093/treephys/tpu101 25536961

[CR3] Asner GP, Martin RE, Knapp DE (2017). Airborne laser-guided imaging spectroscopy to map forest trait diversity and guide conservation. Science.

[CR4] Asuka Y, Tani N, Tsumura Y, Tomaru N (2004) Development and characterization of microsatellite markers for Fagus crenata Blume. Mol Ecol Notes 4(1):101–103. 10.1046/j.1471-8286.2003.00583.x

[CR5] Barzdajn W, Rzeznik Z (2002). Wstepne wyniki miedzynarodowego doswiadczenia proweniencyjnego z bukiem [Fagus sylvatica L.] serii 1993/1995 w Lesnym Zakladzie Doswiadczalnym. Siemnianice.

[CR6] Belletti P, Lanteri S (1996). Allozyme variation among European beech (Fagus sylvatica L.) stands in Piedmont. North-Western Italy Silvae Genet.

[CR7] Bilela S, Dounavi A, Fussi B (2012). Natural regeneration of Fagus sylvatica L. adapts with maturation to warmer and drier microclimatic conditions. For Ecol Manage.

[CR8] Bolte A, Czajkowski T, Kompa T, (2007) The north-eastern distribution range of European beech—a review, Forestry: an Int J Forest Res 80(4):413–429. 10.1093/forestry/cpm028

[CR9] Brun Philipp, Psomas Achilleas, Ginzler Christian (2020). Large-scale early-wilting response of Central European forests to the 2018 extreme drought. Global Change Biology.

[CR10] Capblancq T, Fitzpatrick MC, Bay RA (2020). Genomic prediction of (Mal) adaptation across current and future climatic landscapes. Annu Rev Ecol Evol Syst.

[CR11] Capblancq T, Morin X, Gueguen M (2020). Climate-associated genetic variation in Fagus sylvatica and potential responses to climate change in the French Alps. J Evol Biol.

[CR12] Caudullo G, Welk E, San-Miguel-Ayanz J (2017) Chorological maps for the main European woody species. Data Br 12:. 10.1016/j.dib.2017.05.007 10.1016/j.dib.2017.05.007 PMC5435575 28560272

[CR13] Chmura DJ, Rozkowski R (2002). Variability of beech provenances in spring and autumn phenology. Silvae Genet.

[CR14] Chybicki IJ, Burczyk J (2009). Simultaneous estimation of null alleles and inbreeding coefficients. J Hered.

[CR15] Comps B, Thiébaut B, Paule L (1990). Allozymic variability in beechwoods (Fagus sylvatica l.) over central Europe: spatial differentiation among and within populations. Heredity (edinb).

[CR16] Comps B, Gömöry D, Letouzey J (2001). Diverging trends between heterozygosity and allelic richness during postglacial colonization in the European beech. Genetics.

[CR17] Conkle MT (1992) Genetic diversity—seeing the forest through the trees. 5–22. 10.1007/978-94-011-2815-5_3

[CR18] Cuervo-Alarcon L, Arend M, Müller M, et al (2018) Genetic variation and signatures of natural selection in populations of European beech (Fagus sylvatica L.) along precipitation gradients. Tree Genet Genomes 14:. 10.1007/s11295-018-1297-2

[CR19] Cuervo- Alarcon L, Arend M, Müller M, Sperisen C, Finkeldey R, Krutovsky KV (2021) A candidate gene association analysis iden- tifies SNPs potentially involved in drought tolerance in European beech (Fagus sylvatica L.). Sci Rep 11(1):2386. 10.1038/s4159-021-81594-w 10.1038/s41598-021-81594-w PMC7840767 33504857

[CR20] Cvrčková H, Máchová P, Poláková L, Trčková O (2017). Evaluation of the genetic diversity of selected Fagus sylvatica L populations in the Czech Republic using nuclear microsatellites. J For Sci.

[CR21] Danecek P, Auton A, Abecasis G (2011). The variant call format and VCFtools. Bioinformatics.

[CR22] De Lafontaine G, Ducousso A, Lefèvre S (2013). Stronger spatial genetic structure in recolonized areas than in refugia in the European beech. Mol Ecol.

[CR23] Demesure B, Comps B, Petit RJ (1996). Chloroplast DNA phylogeography of the common beech (Fagus sylvatica L.) in Europe. Evolution (N Y).

[CR24] Dinno A (2017) dunn.test: Dunn's test of multiple comparisons using rank sums_. R package version 1.3.5

[CR25] Dittmar Christoph, Elling Wolfram (2006). Phenological phases of common beech (Fagus sylvatica L) and their dependence on region and altitude in southern germany. European Journal of Forest Research.

[CR26] Dounavi A, Koutsias N, Ziehe M, Hattemer HH (2010). Spatial patterns and genetic structures within beech populations (Fagus sylvatica L.) of forked and non-forked individuals. Eur J for Res.

[CR27] Dunnington D (2020) ggspatial: Spatial Data Framework for ggplot2

[CR28] Duputié A, Rutschmann A, Ronce O, Chuine I (2015). Phenological plasticity will not help all species adapt to climate change. Glob Chang Biol.

[CR29] Dyderski MK, Paź S, Frelich LE, Jagodziński AM (2018) How much does climate change threaten European forest tree species distributions? Glob Chang Biol 24:. 10.1111/gcb.13925 10.1111/gcb.13925 28991410

[CR30] Ellegren H, Galtier N (2016) Determinants of genetic diversity. Nat Rev Genet 17:422–433 10.1038/nrg.2016.58 27265362

[CR31] Elleouet JS, Aitken SN (2019) Long-distance pollen dispersal during recent colonization favors a rapid but partial recovery of genetic diversity in Picea sitchensis. New Phytol 222:. 10.1111/nph.15615 10.1111/nph.15615 30485444

[CR32] Emiliani G, Paffetti D, Vettori C, Giannini R (2004). Geographic distribution of genetic variability of Fagus sylvatica L. in Southern Italian populations. For Genet.

[CR33] Excoffier L, Foll M, Petit RJ (2009) Genetic consequences of range expansions. Annu Rev Ecol Evol Syst 40:. 10.1146/annurev.ecolsys.39.110707.173414

[CR34] Falk W, Hempelmann N (2013). Species favourability shift in Europe due to climate change: a case study for Fagus sylvatica L. and Picea abies (L.) Karst Based on an Ensemble of Climate Models. J Climatol.

[CR35] Fang J, Lechowicz MJ (2006). Climatic limits for the present distribution of beech (Fagus L) species in the world. J Biogeogr.

[CR36] Garnier S (2018). viridis: default color maps from 'matplotlib'. R package version 0.5.1. https://CRAN.R-project.org/package=viridis

[CR37] Gauzere J, Klein E, Oddou-Muratorio S (2013) Ecological determinants of mating system within and between three Fagus sylvatica populations along an elevational gradient. Mol Ecol 22:5001–5015 10.1111/mec.12435 23952125

[CR38] Giesecke Thomas, Hickler Thomas, Kunkel Timo (2007). Towards an understanding of the Holocene distribution of Fagus sylvatica L. Journal of Biogeography.

[CR39] Gömöry D, Hynek V, Paule L (1998). Delineation of seed zones for European beech (Fagus sylvatica L.) in the Czech Republic based on isozyme gene markers. Ann Des Sci for.

[CR40] Guichoux E, Lagache L, Wagner S (2011). Current trends in microsatellite genotyping. Mol Ecol Resour.

[CR41] Hatziskakis S, Papageorgiou AC, Gailing O, Finkeldey R (2009). High chloroplast haplotype diversity in Greek populations of beech (Fagus sylvatica L.). Plant Biol.

[CR42] Hijmans R (2022) geosphere: spherical trigonometry. R package version 1.5-18

[CR43] Hijmans R (2020) raster: geographic analysis and modeling with raster data. R package version 31–5

[CR44] Jiménez-Alfaro B, Girardello M, Chytrý M (2018). History and environment shape species pools and community diversity in European beech forests. Nat Ecol Evol.

[CR45] Jombart T (2008). Adegenet: A R package for the multivariate analysis of genetic markers. Bioinformatics.

[CR46] Jump AS, Hunt JM, Pen̈uelas J,  (2006). Rapid climate change-related growth decline at the southern range edge of Fagus sylvatica. Glob Chang Biol.

[CR47] Jump AS, Rico L, Coll M, Peñuelas J (2012) Wide variation in spatial genetic structure between natural populations of the European beech (Fagus sylvatica) and its implications for SGS comparability. Heredity 108(6):633–639. 10.1038/hdy.2012.1 10.1038/hdy.2012.1 PMC3356813 22354112

[CR48] Kassambara A (2022) rstatix: pipe-friendly framework for basic statistical tests. R package version 0.7.1,

[CR49] Kassambara A (2022) ggpubr: 'ggplot2' Based Publication Ready Plots. R package version 0.5.0

[CR50] Kempf M., Konnert M. (2016). Distribution of genetic diversity in Fagus sylvatica at the north-eastern edge of the natural range. Silva Fennica vol. 50 no. 4 article id 1663-17 10.14214/sf.1663

[CR51] Kolde R (2019) pheatmap: pretty heatmaps R package version 1.0.12

[CR52] Kramer K, Degen B, Buschbom J (2010). Modelling exploration of the future of European beech (Fagus sylvatica L.) under climate change-range, abundance, genetic diversity and adaptive response. For Ecol Manage.

[CR53] Kreyling J, Thiel D, Nagy L (2012). Late frost sensitivity of juvenile Fagus sylvatica L differs between southern Germany and Bulgaria and depends on preceding air temperature. Eur J For Res.

[CR54] Lalagüe H., Csilléry K., Oddou-Muratorio S., Safrana J., de Quattro C., Fady B., González-Martínez S. C., Vendramin G. G. (2014). Nucleotide diversity and linkage disequilibrium at 58 stress response and phenology candidate genes in a European beech (Fagus sylvatica L.) population from southeastern France. Tree Genetics & Genomes.

[CR55] Lander TA, Oddou-Muratorio S, Prouillet-Leplat H, Klein EK (2011). Reconstruction of a beech population bottleneck using archival demographic information and Bayesian analysis of genetic data. Mol Ecol.

[CR56] Lander TA, Klein EK, Roig A (2021) Weak founder effects but significant spatial genetic imprint of recent contraction and expansion of European beech populations. Heredity (Edinb) 491–504. 10.1038/s41437-020-00387-5 10.1038/s41437-020-00387-5 PMC8027192 33230286

[CR57] Landguth EL, Fedy BC, Oyler-Mccance SJ (2012). Effects of sample size, number of markers, and allelic richness on the detection of spatial genetic pattern. Mol Ecol Resour.

[CR58] Leeper TJ (2018) tabulizer: Bindings for Tabula PDF Table Extractor Library. R package version 0.2.3

[CR59] LEFÈVRE S., WAGNER S., PETIT R. J., De LAFONTAINE G. (2012). Multiplexed microsatellite markers for genetic studies of beech. Molecular Ecology Resources.

[CR60] Lesser MR, Parchman TL, Jackson ST (2013) Development of genetic diversity, differentiation and structure over 500 years in four ponderosa pine populations. Mol Ecol 22:. 10.1111/mec.12280 10.1111/mec.12280 23495837

[CR61] Lesur I, Bechade A, Lalanne C, Klopp C, Noirot C, Leplé J- C, Le Provost G (2015) A unigene set for European beech (Fagus sylvat- ica L.) and its use to decipher the molecular mechanisms involved in dormancy regulation. Mol Ecol Resour 15(5):1192– 1204. 10.1111/1755-0998.12373 10.1111/1755-0998.12373 25594128

[CR62] Mader M, Liesebach H, Liesebach M, Kersten B (2019). The complete chloroplast genome sequence of Fagus sylvatica L. (Fagaceae). Mitochondrial DNA Part B Resour.

[CR63] Magri D (2008). Patterns of post-glacial spread and the extent of glacial refugia of European beech (Fagus sylvatica). J Biogeogr.

[CR64] Magri D, Vendramin GG, Comps B (2006). A new scenario for the Quaternary history of European beech populations: palaeobotanical evidence and genetic consequences. New Phytol.

[CR65] Meger J, Ulaszewski B, Vendramin GG, Burczyk J (2019) Using reduced representation libraries sequencing methods to identify cpDNA polymorphisms in European beech (Fagus sylvatica L). Tree Genet Genomes 15:. 10.1007/s11295-018-1313-6

[CR66] Merzeau D, Di Giusto F, Comps B, et al (1989) Genetic control of isozyme systems and heterogeneity of pollen contribution in beech (Fagus sylvatica L.). Silvae Genetica 38:0037–5349

[CR67] Metzker ML (2010) Sequencing technologies the next generation. Nature Review Genetics 11(1):31–46. 10.1038/nrg2626 10.1038/nrg2626 19997069

[CR68] Millard SP (2013) EnvStats: an R package for environmental statistics. Springer, New York

[CR69] Mishra B, Ulaszewski B, Ploch S (2021). A circular chloroplast genome of fagus sylvatica reveals high conservation between two individuals from Germany and one individual from Poland and an alternate direction of the small single-copy region. Forests.

[CR70] Mishra B, Ulaszewski B, Meger J, et al (2021a) A comparison of three circular mitochondrial genomes of fagus sylvatica from germany and poland reveals low variation and complete identity of the gene space. Forests 12:571. 10.3390/f12050571

[CR71] Mishra B, Ulaszewski B, Meger J, et al. (2022) A chromosome-level genome assembly of the european beech (fagus sylvatica) reveals anomalies for organelle DNA integration, repeat content and distribution of SNPs. Front Genet 8;12:691058. 10.3389/fgene.2021.691058 10.3389/fgene.2021.691058 PMC8862710 35211148

[CR72] Müller-Starck R (1996) Genetische Aspekte der Reproduktion der Buche (Fagus sylvatica L.) unter Berücksichtigung waldbaulicher Gegebenheiten. na

[CR73] Muffler L, Weigel R, Hacket-Pain AJ (2020). Lowest drought sensitivity and decreasing growth synchrony towards the dry distribution margin of European beech. J Biogeogr.

[CR74] Müller M, Cuervo-Alarcon L, Gailing O (2018). Genetic variation of european beech populations and their progeny from northeast Germany to Southwest Switzerland. Forests.

[CR75] Nekrutenko A, Taylor J (2012) Next-generation sequencing data interpretation: enhancing reproducibility and accessibility. Nature Review Genetics 13, 667–672. 10.1038/nrg3305 10.1038/nrg3305 22898652

[CR76] Neuwirth E (2014) RColorBrewer: ColorBrewer palettes. R Package version 1.2-2

[CR77] Nowakowska, JA, Sułkowska, M (2011) Genetic structure of European beech of mother and progeny stands in Poland on the basis of DNA chloroplast markers.

[CR78] Oddou-Muratorio S, Gauzere J, Bontemps A, Rey J, Klein EK (2018) Fagus sylvatica. Molecular Ecology 27(15):3131–3145. 10.1111/mec.14770 10.1111/mec.14770 29924889

[CR79] Paffetti D, Travaglini D, Buonamici A (2012). The influence of forest management on beech (Fagus sylvatica L.) stand structure and genetic diversity. For Ecol Manage.

[CR80] Paradis E (2010) Pegas: an R package for population genetics with an integrated-modular approach. Bioinformatics 26(3):419–420. 10.1093/bioinformatics/btp696 10.1093/bioinformatics/btp696 20080509

[CR81] Pastorelli R, Smulders MJM, Van’t Westende WPC, Vosman B, Giannini R, Vettori C, Vendramin GG (2003) Characterization of microsatellite markers in Fagus sylvatica L. and Fagus orientalis Lipsky. Molecular Ecology Notes 3:76–78. 10.1046/j.1471-8286.2003.00355.x

[CR82] Pebesma EJ (2004). Multivariable geostatistics in S: The gstat package. Comput Geosci.

[CR83] Pebesma E (2018) Simple features for R: Standardized support for spatial vector data. R J. 10.32614/rj-2018-009

[CR84] Peterson BK, Weber JN, Kay EH, Fisher HS, Hoekstra HE, Orlando L (2012) Double digest RADseq: An inexpensive method for De Novo SNP discovery and genotyping in model and non-model species. PLoS ONE 7(5):e37135. 10.1371/journal.pone.0037135 10.1371/journal.pone.0037135 PMC3365034 22675423

[CR85] Petit RJ, Aguinagalde I, De Beaulieu JL (2003). Glacial refugia: hotspots but not melting pots of genetic diversity. Science.

[CR86] Pfenninger M, Reuss F, Kiebler A, et al (2021) Genomic basis of drought resistance in Fagus sylvatica. bioRxiv 1–21. 10.1101/2020.12.04.411264

[CR87] Piotti A, Leonardi S, Buiteveld J, Geburek T, Gerber S, Kramer K, Vettori C, Vendramin GG (2012) Comparison of pollen gene flow among four European beech (Fagus sylvatica L.) populations characterized by different management regimes. Heredity 108(3):322–331. 10.1038/hdy.2011.77 10.1038/hdy.2011.77 PMC3282401 21897442

[CR88] Pluess AR, Frank A, Heiri C (2016). Genome-environment association study suggests local adaptation to climate at the regional scale in Fagus sylvatica. New Phytol.

[CR89] Pluess AR (2011) Pursuing glacier retreat: Genetic structure of a rapidly expanding Larix decidua population. Mol Ecol 20:. 10.1111/j.1365-294X.2010.04972.x 10.1111/j.1365-294X.2010.04972.x 21199030

[CR90] Postolache D, Oddou-Muratorio S, Vajana E (2021). Genetic signatures of divergent selection in European beech ( Fagus sylvatica L.) are associated with the variation in temperature and precipitation across its distribution range. Mol Ecol.

[CR91] Príncipe A, van der Maaten E, van der Maaten-Theunissen M (2017). Low resistance but high resilience in growth of a major deciduous forest tree (Fagus sylvatica L.) in response to late spring frost in southern Germany. Trees - Struct Funct.

[CR92] Pullin AS, Stewart GB (2006). Guidelines for systematic review in conservation and environmental management. Conserv Biol.

[CR93] R Core Team (2020) (2020) R: A language and environment for statistical computing. R A Lang Environ Stat Comput R Found Stat Comput Vienna, Austria

[CR94] Reddy SB, Rosenberg NA (2012). Refining the relationship between homozygosity and the frequency of the most frequent allele. J Math Biol.

[CR95] Refaeilzadeh P, Tang L, Liu H (2009) Cross-validation. In: LIU L, ÖZSU MT (eds) Encyclopedia of Database Systems. Springer US, Boston, MA, pp 532–538

[CR96] Rose L, Leuschner C, Köckemann B, Buschmann H (2009). Are marginal beech (Fagus sylvatica L.) provenances a source for drought tolerant ecotypes?. Eur J for Res.

[CR97] Rosenberg Noah A, Jakobsson Mattias (2008) The Relationship Between Homozygosity and the Frequency of the Most Frequent Allele. Genetics 179(4):2027–2036. 10.1534/genetics.107.084772 10.1534/genetics.107.084772 PMC2516077 18689892

[CR98] Rstudio Team (2019) RStudio: Integrated development for R. RStudio, Inc., Boston MA. RStudio

[CR99] Saltré F, Saint-Amant R, Gritti ES (2013). Climate or migration: what limited European beech post-glacial colonization?. Glob Ecol Biogeogr.

[CR100] Saltré F, Duputié A, Gaucherel C, Chuine I (2015). How climate, migration ability and habitat fragmentation affect the projected future distribution of European beech. Glob Chang Biol.

[CR101] Sander T, König S, Rothe GM (2000). Genetic variation of European beech (Fagus sylvatica L.) along an altitudinal transect at mount Vogelsberg in Hesse. Germany Mol Ecol.

[CR102] Sangüesa-Barreda G, Di Filippo A, Piovesan G (2021). Warmer springs have increased the frequency and extension of late-frost defoliations in southern European beech forests. Sci Total Environ.

[CR103] Schuldt B, Buras A, Arend M (2020). A first assessment of the impact of the extreme 2018 summer drought on Central European forests. Basic Appl Ecol.

[CR104] Shi MM, Chen XY (2012) Leading-edge populations do not show low genetic diversity or high differentiation in a wind-pollinated tree. Popul Ecol 54:. 10.1007/s10144-012-0332-7

[CR105] Silva E, Rezende D, Mazzella P, Legay M (2012). Does natural regeneration determine the limit of European beech distribution under climatic stress?. For Ecol Manage.

[CR106] Sjolund JM, Gonzalez-Diaz P, Moreno-Villena JJ, Jump AS (2017). Understanding the Legacy of Widespread Population Translocations on the Post-Glacial Genetic Structure of the.

[CR107] Sjölund MJ, Jump AS (2015). Coppice management of forests impacts spatial genetic structure but not genetic diversity in European beech (Fagus sylvatica L.). For Ecol Manage.

[CR108] Smulders MJM, Cottrell JE, Lefèvre F, et al (2008) Structure of the genetic diversity in black poplar (Populus nigra L.) populations across European river systems: consequences for conservation and restoration. For Ecol Manag 255:. 10.1016/j.foreco.2007.10.063

[CR109] Stefano L, Piovani P, Scalfi M, Piotti A, Giannini R, Menozzi P (2012) Effect of habitat fragmentation on the genetic diversity and structure of peripheral populations of beech in central Italy. J Hered 103(3):408–417. 10.1093/jhered/ess004 10.1093/jhered/ess004 22496339

[CR110] Taberlet P, Gielly L, Pautou G, Bouvet J (1991). Universal primers for amplification of three non-coding regions of chloroplast DNA. Plant Mol Biol.

[CR111] Teixeira JC, Huber CD (2021) The inflated significance of neutral genetic diversity in conservation genetics. Proc Natl Acad Sci 118:. 10.1073/PNAS.2015096118 10.1073/pnas.2015096118 PMC7958437 33608481

[CR112] Tennekes M (2018) Tmap: Thematic maps in R. J Stat Softw 84:. 10.18637/jss.v084.i06

[CR113] Thiebaut B, Vernet P, Lumaret R (1982). The bud enzymes of beech (Fagus sylvatica L.) Genetic distinction and analysis of polymorphism in several french populations. Silvae Genet.

[CR114] Ueno Saneyoshi, Taguchi Yuriko, Tomaru Nobuhiro, Tsumura Yoshihiko (2009). Development of EST-SSR markers from an inner bark cDNA library of Fagus crenata (Fagaceae). Conservation Genetics.

[CR115] Ulaszewski B, Meger J, Burczyk J (2021). Comparative analysis of SNP discovery and genotyping in Fagus sylvatica L. and Quercus robur L. using RADseq, GBS, and ddRAD methods. Forests.

[CR116] Varsamis G, Papageorgiou AC, Merou T (2019). Adaptive diversity of beech seedlings under climate change scenarios. Front Plant Sci.

[CR117] Vettori C, Vendramin GG, Anzidei M (2004). Geographic distribution of chloroplast variation in Italian populations of beech (Fagus sylvatica L.). Theor Appl Genet.

[CR118] Vitasse Y, Bottero A, Cailleret M (2019). Contrasting resistance and resilience to extreme drought and late spring frost in five major European tree species. Glob Chang Biol.

[CR119] Vos P, Hogers R, Bleeker M (1995). AFLP: a new technique for DNA fingerprinting. Nucleic Acids Res.

[CR120] Wang KS (2003). Genetic diversity and temporal genetic structure in European beech (Fagus sylvatica L.). Silvae Genet.

[CR121] Wang KS (2004). Gene flow in European beech (Fagus sylvatica L.). Genetica.

[CR122] Wang DR, Venturas MD, Mackay DS (2020). Use of hydraulic traits for modeling genotype-specific acclimation in cotton under drought. New Phytol.

[CR123] Wang WT, Xu B, Zhang DY, Bai WN (2016) Phylogeography of postglacial range expansion in Juglans mandshurica (Juglandaceae) reveals no evidence of bottleneck, loss of genetic diversity, or isolation by distance in the leading-edge populations. Mol Phylogenet Evol 102:. 10.1016/j.ympev.2016.06.005 10.1016/j.ympev.2016.06.005 27346642

[CR124] Weising K, Gardner RC (1999). A set of conserved PCR primers for the analysis of simple sequence repeat polymorphisms in chloroplast genomes of dicotyledonous angiosperms. Genome.

[CR125] Wickham H, Henry L (2019) tidyr: Tidy Messy Data. R Package version 1.0.0

[CR126] Wickham H, Averick M, Bryan J, et al (2019) Welcome to the Tidyverse. Journal of Open Source Software 10.21105/joss.01686

[CR127] Wickham, H (2016) Ggplot2: Elegant graphics for data analysis (2nd ed.) Springer International Publishing

[CR128] Wilke C (2016) cowplot: Streamlined plot theme and plot annotations for “ggplot2”. R package version 0.7.0. https://CRAN.R-project.org/package=cowplot

[CR129] Wilke C (2022) ggridges: Ridgeline Plots in 'ggplot2'. R package version 0.5.4, https://wilkelab.org/ggridges/

[CR130] Wilson EO (1988) Biodiversity. National Academy Press, Washington, D.C

[CR131] Yıldırım Y, Tinnert J, Forsman A (2018). Contrasting patterns of neutral and functional genetic diversity in stable and disturbed environments. Ecol Evol.

[CR132] Zeng X, Fischer GA (2021) Using multiple seedlots in restoration planting enhances genetic diversity compared to natural regeneration in fragmented tropical forests. For Ecol Manag 482:. 10.1016/j.foreco.2020.118819

