# Supplementary figures and images for: A novel synthesis of two decades of microsatellite studies on European beech reveals decreasing genetic diversity from glacial refugia
Source: Tree Genet Genomes. 2022 Dec 12;19(1):3. doi: 10.1007/s11295-022-01577-4 (PMC9744708; doi:10.1007/s11295-022-01577-4)

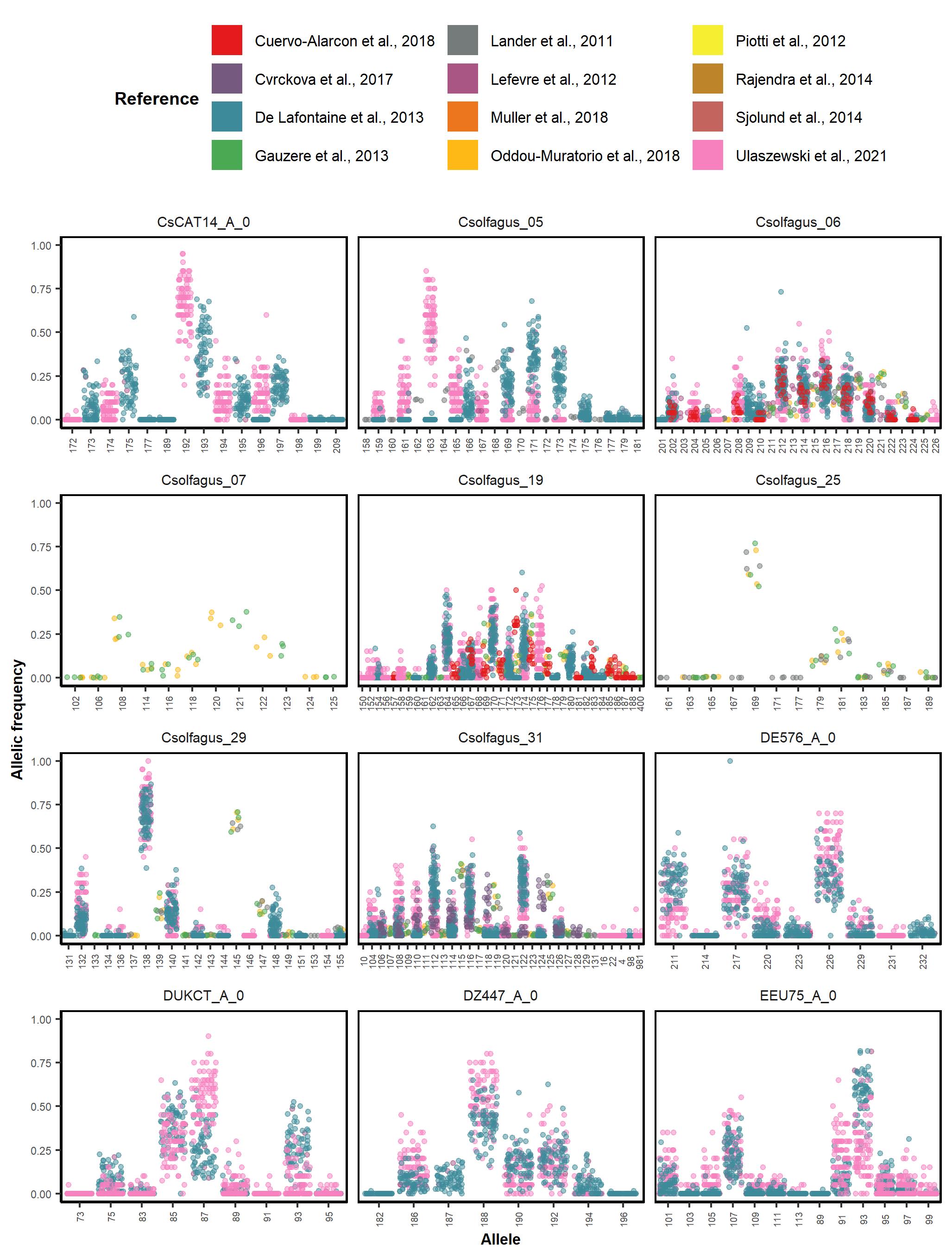

Supplement: Supplementary file 1 — Supplementary file1 (JPG 511 KB) [file 11295_2022_1577_MOESM1_ESM.jpg]

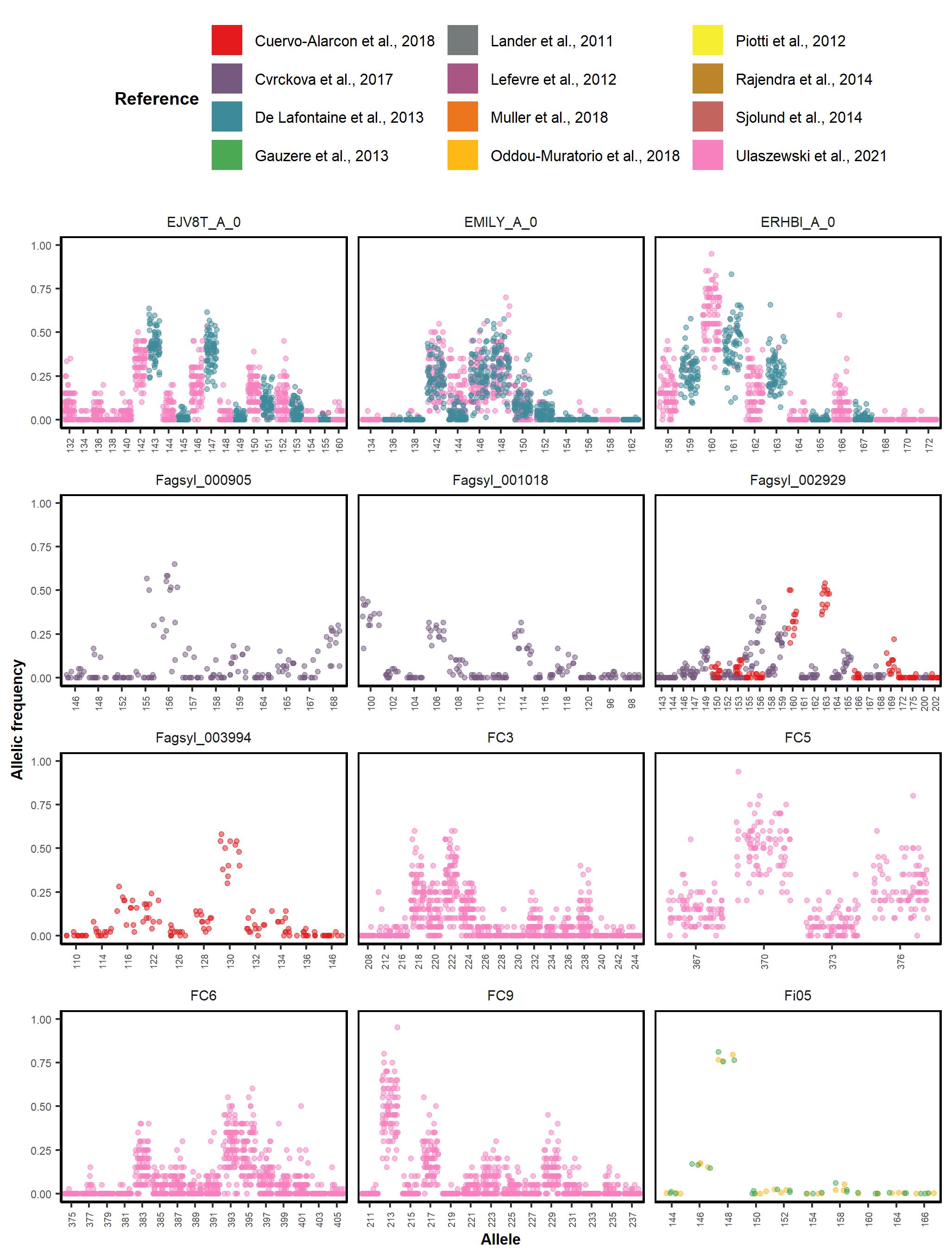

Supplement: Supplementary file 2 — Supplementary file2 (JPG 454 KB) [file 11295_2022_1577_MOESM2_ESM.jpg]

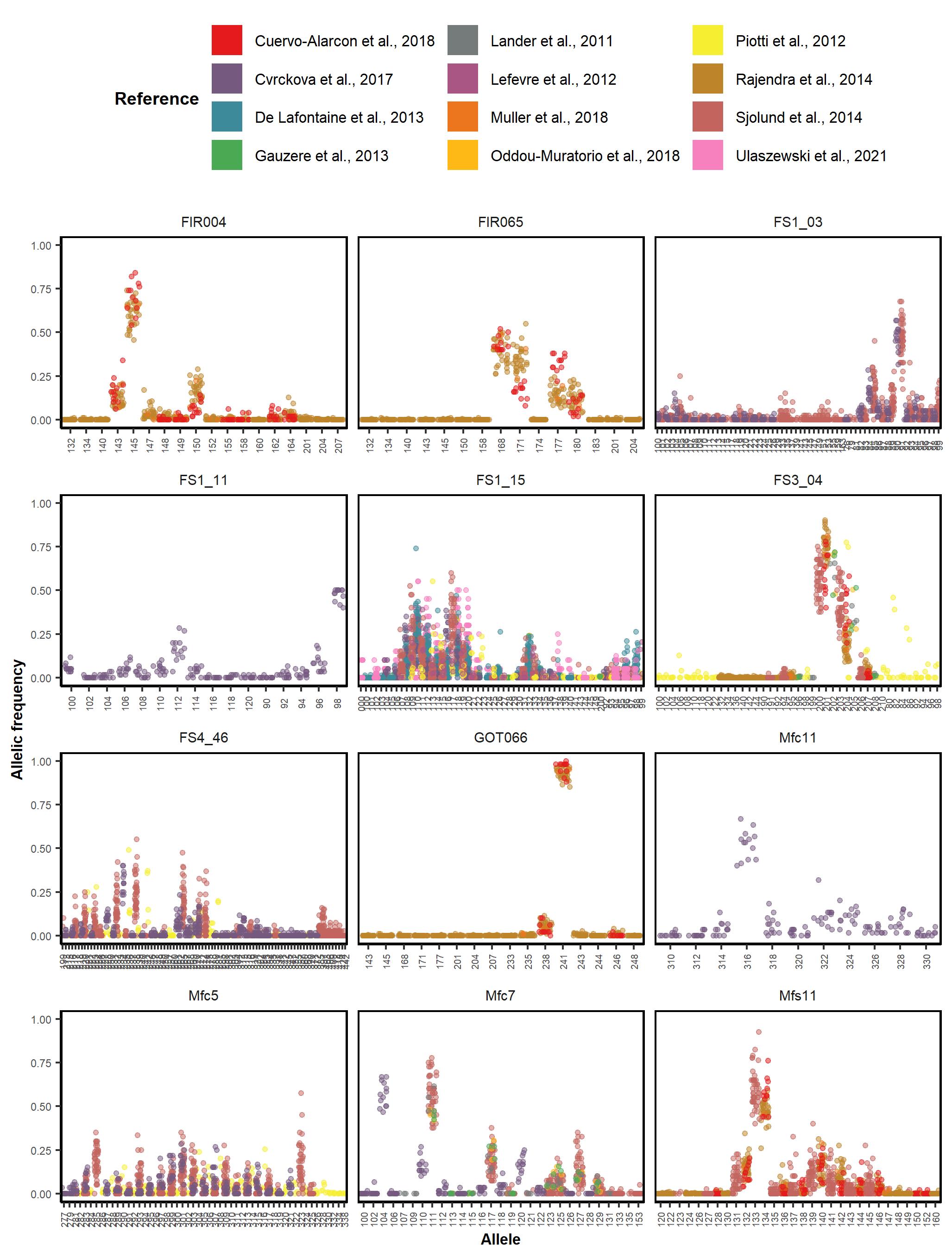

Supplement: Supplementary file 3 — Supplementary file3 (JPG 486 KB) [file 11295_2022_1577_MOESM3_ESM.jpg]

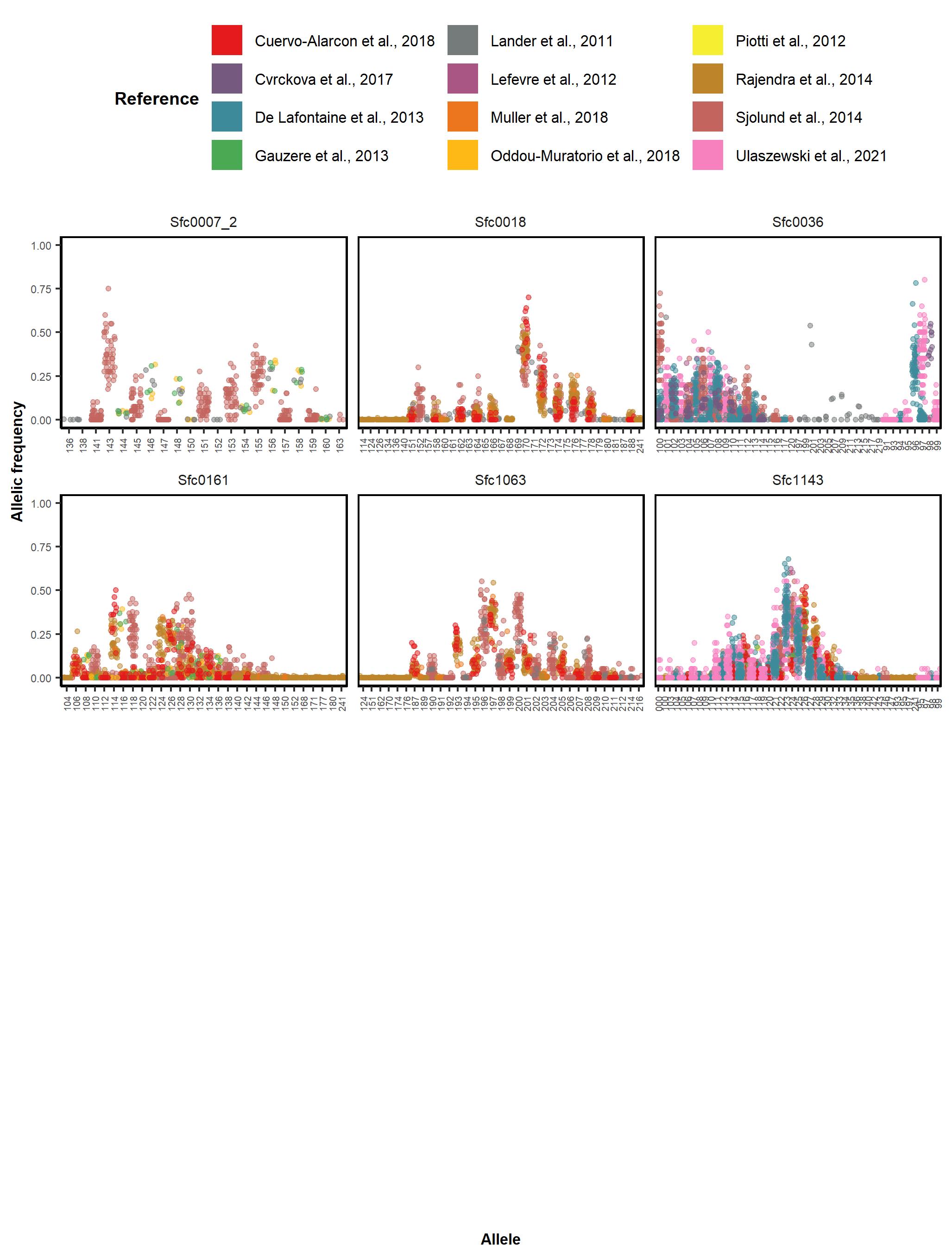

Supplement: Supplementary file 4 — Supplementary file4 (JPG 343 KB) [file 11295_2022_1577_MOESM4_ESM.jpg]

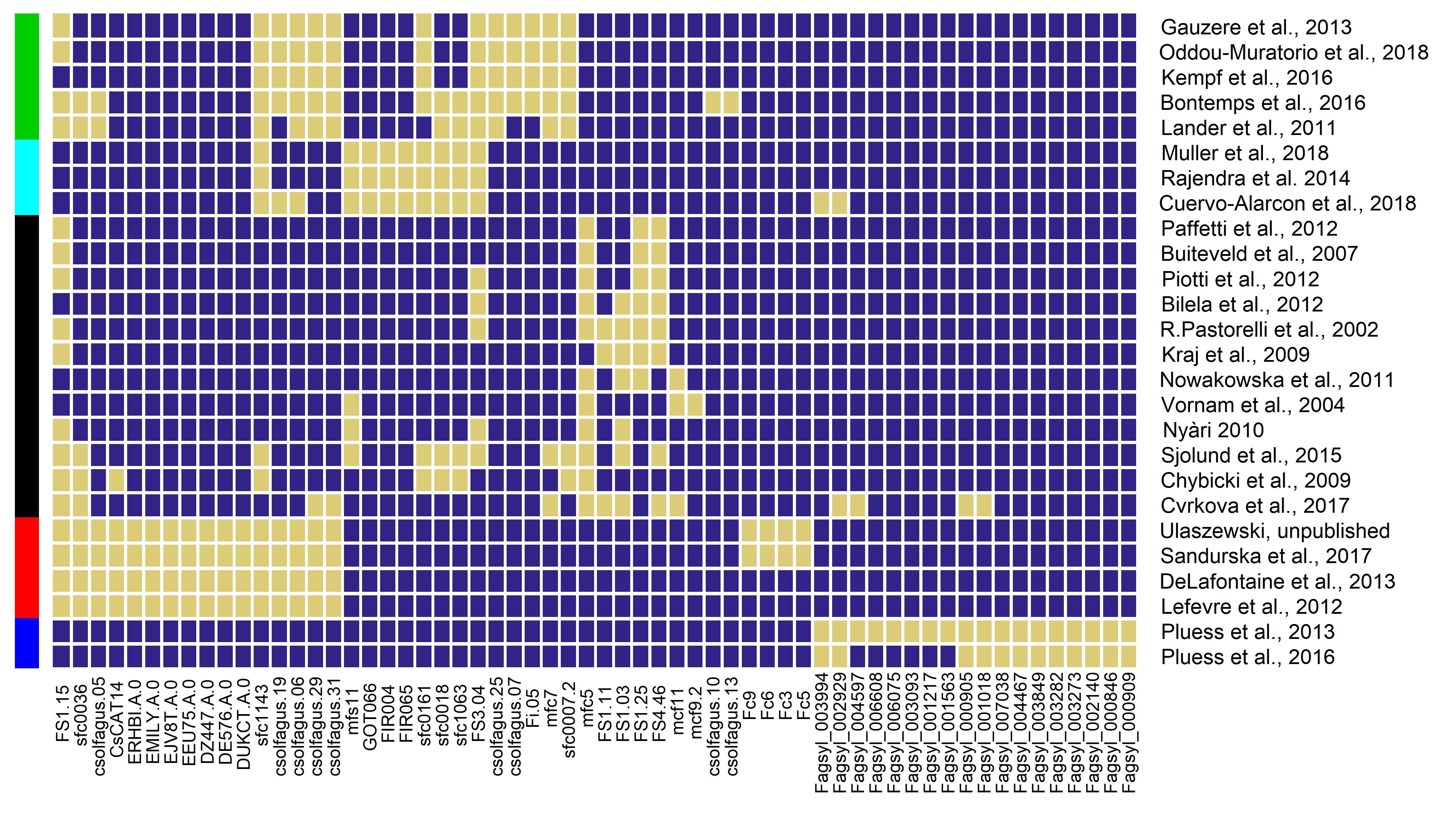

Supplement: Supplementary file 5 — Supplementary file5 (JPG 3126 KB) [file 11295_2022_1577_MOESM5_ESM.jpg]

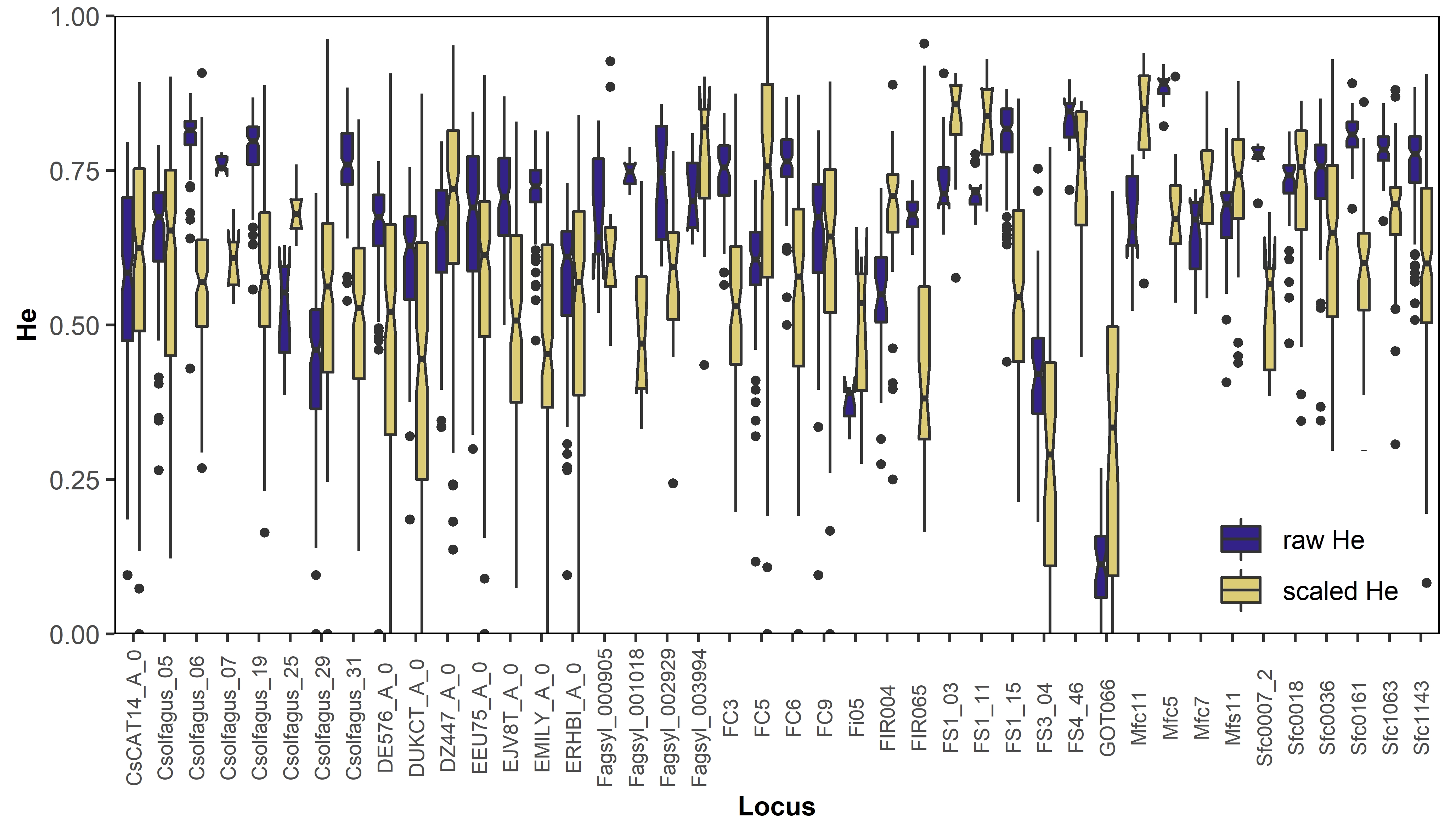

Supplement: Supplementary file 6 — Supplementary file6 (JPG 1651 KB) [file 11295_2022_1577_MOESM6_ESM.jpg]

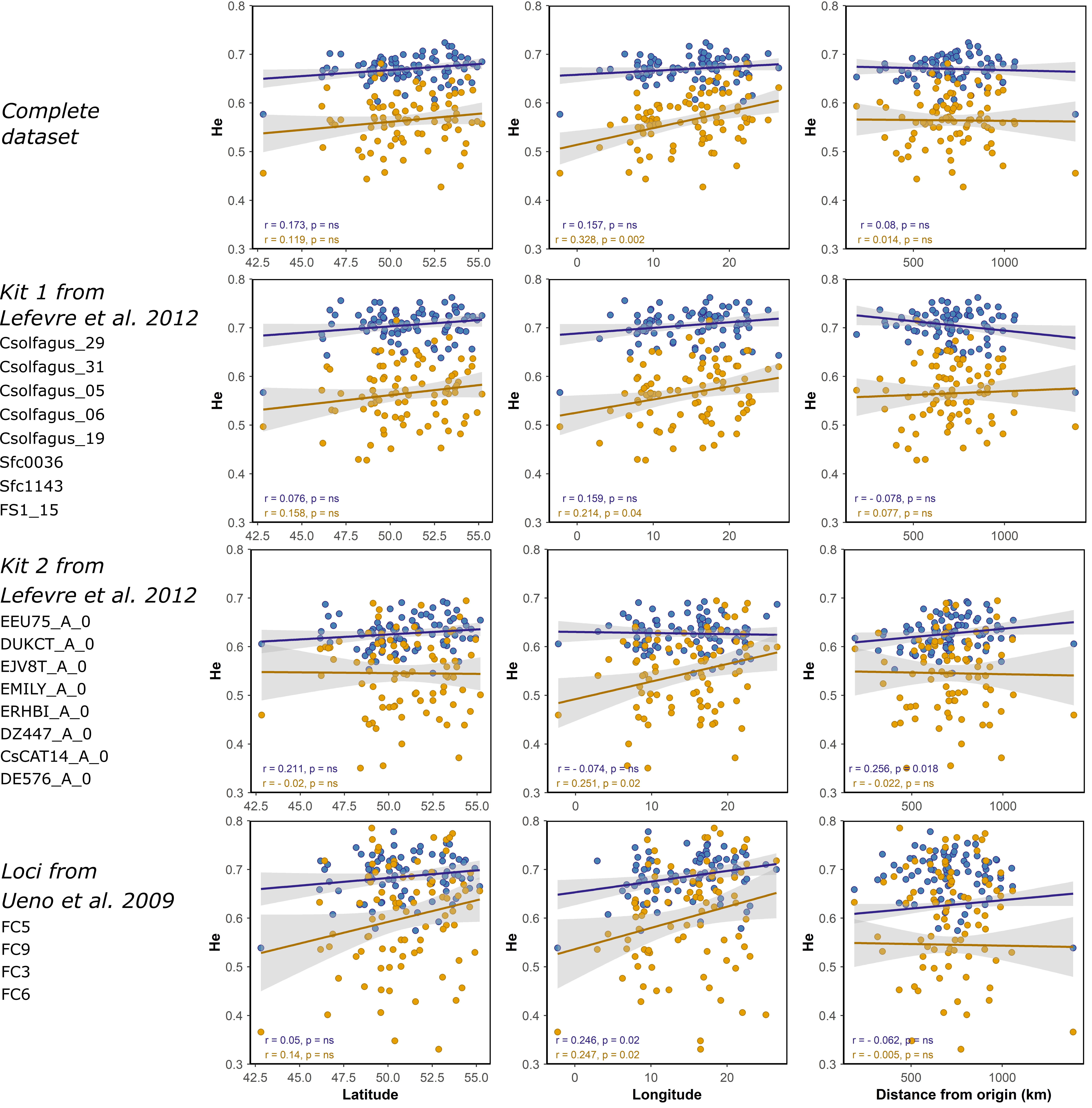

Supplement: Supplementary file 7 — Supplementary file7 (PNG 7402 KB) [file 11295_2022_1577_MOESM7_ESM.png]

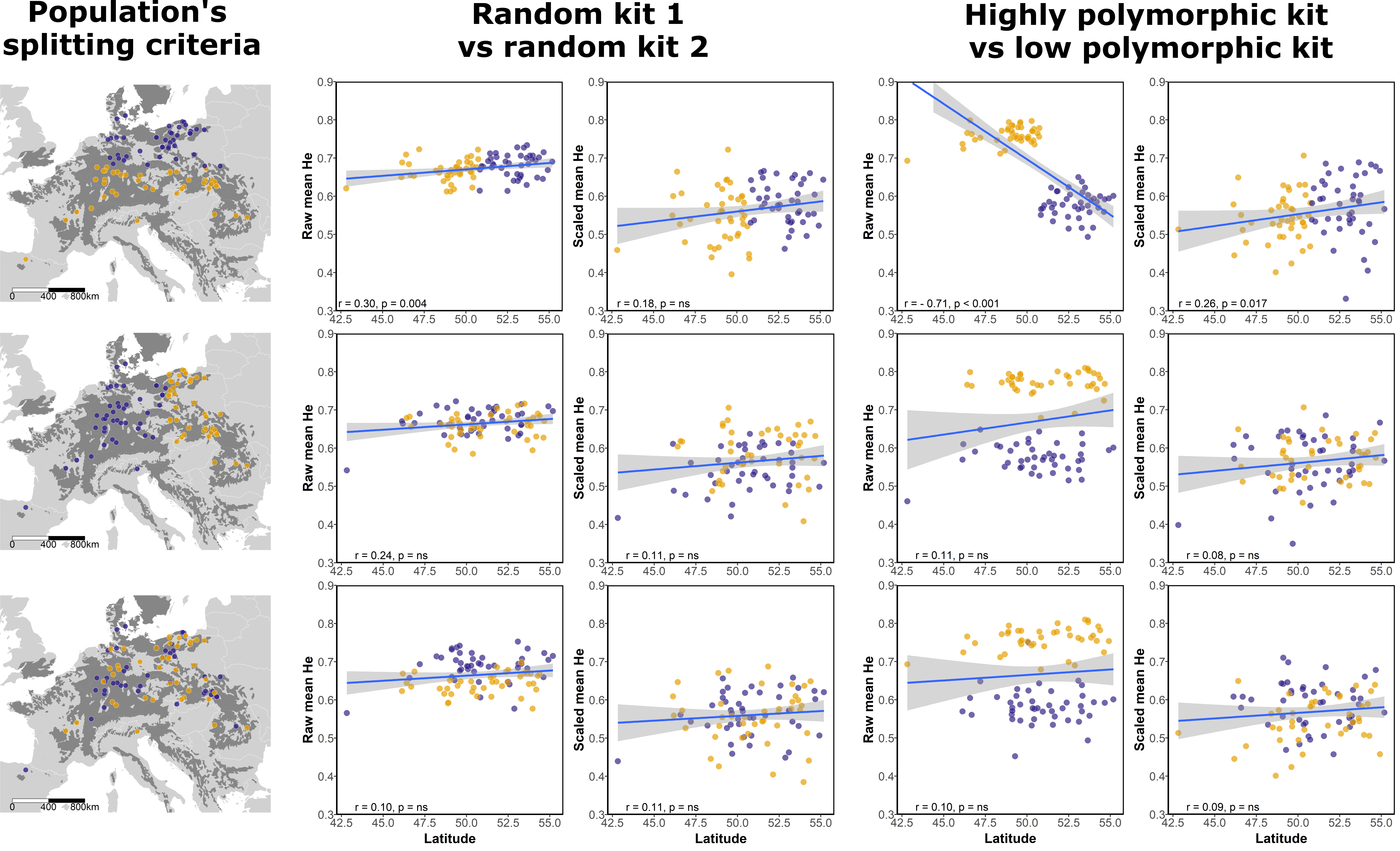

Supplement: Supplementary file 8 — Supplementary file8 (PNG 4572 KB) [file 11295_2022_1577_MOESM8_ESM.png]

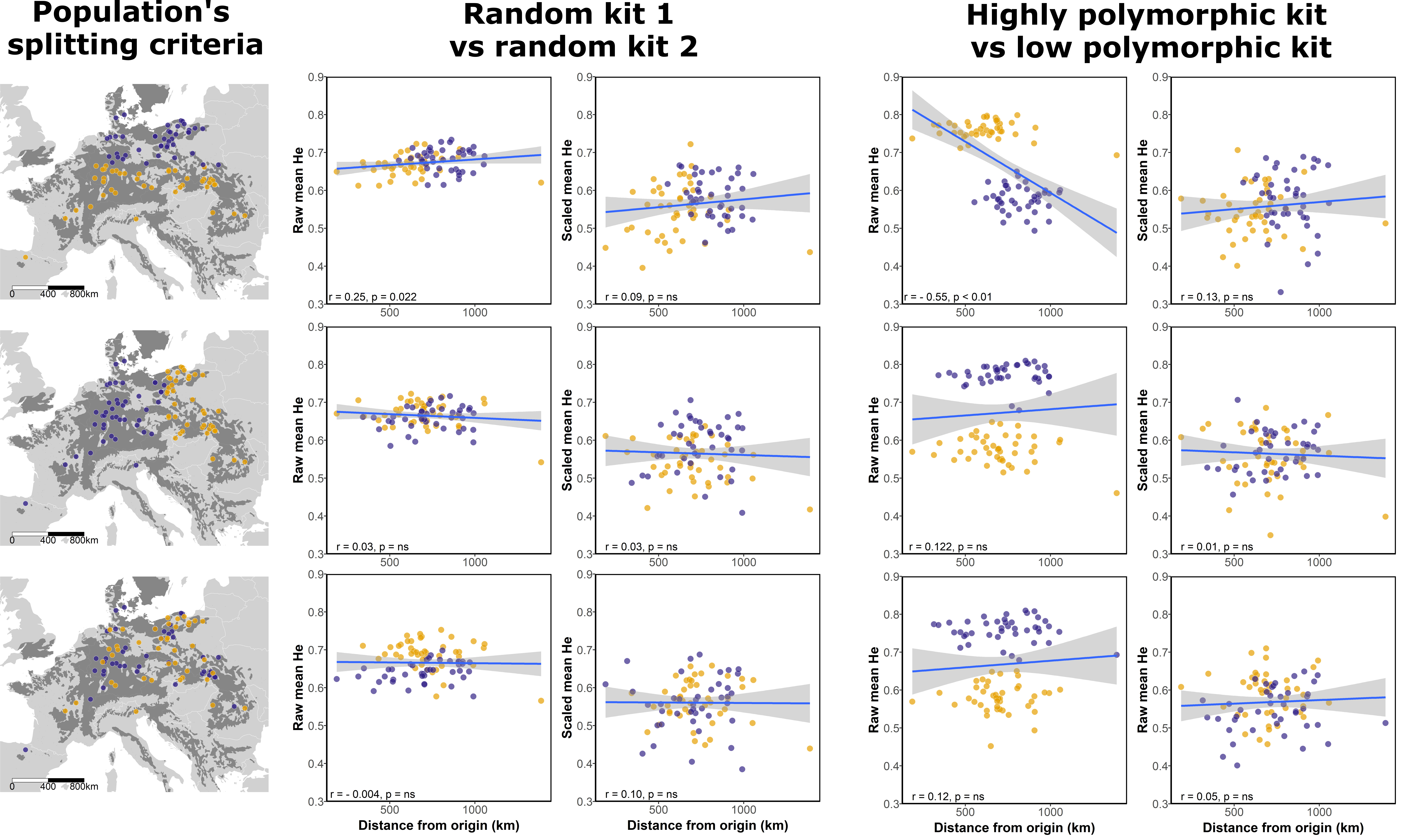

Supplement: Supplementary file 9 — Supplementary file9 (PNG 4371 KB) [file 11295_2022_1577_MOESM9_ESM.png]

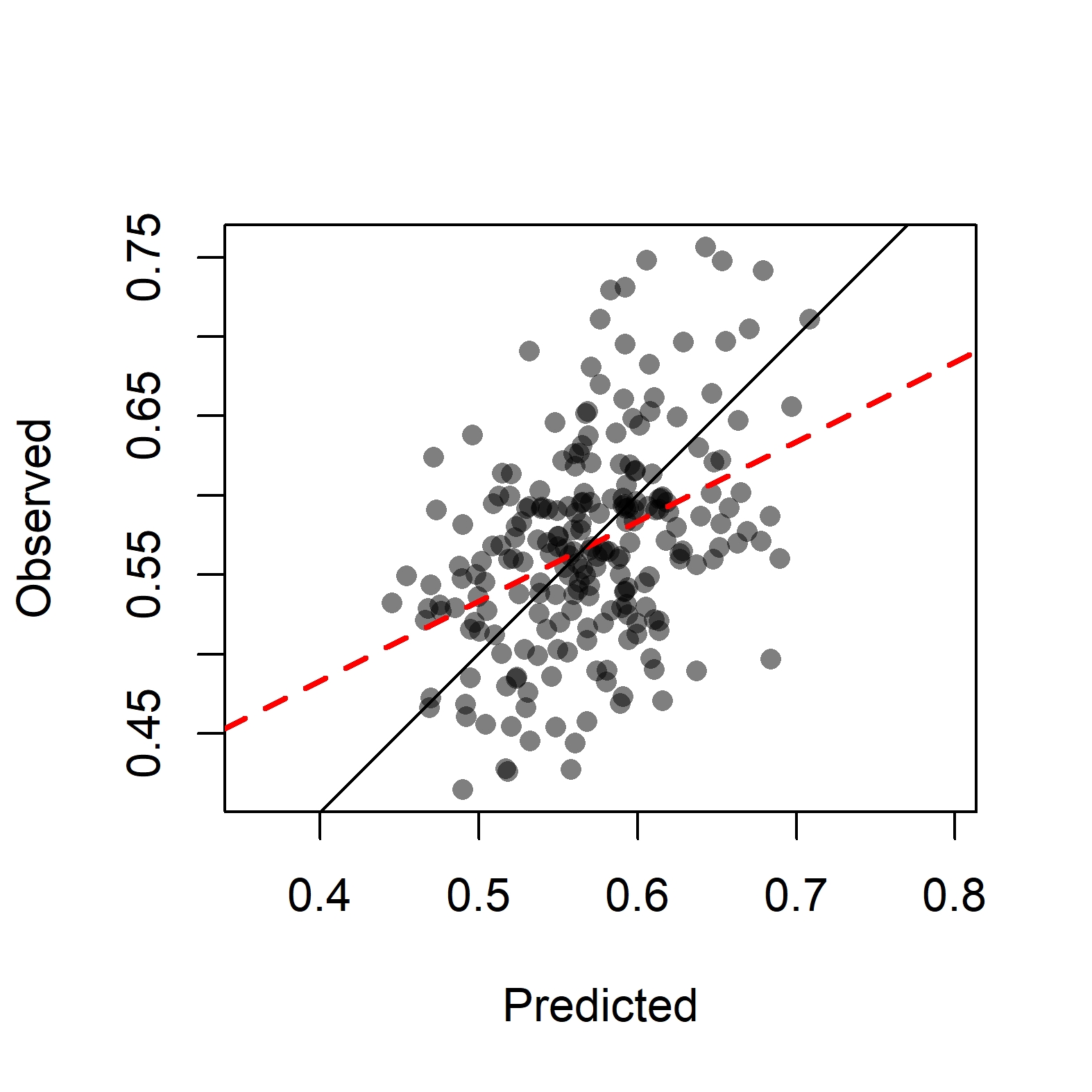

Supplement: Supplementary file 10 — Supplementary file10 (JPG 336 KB) [file 11295_2022_1577_MOESM10_ESM.jpg]

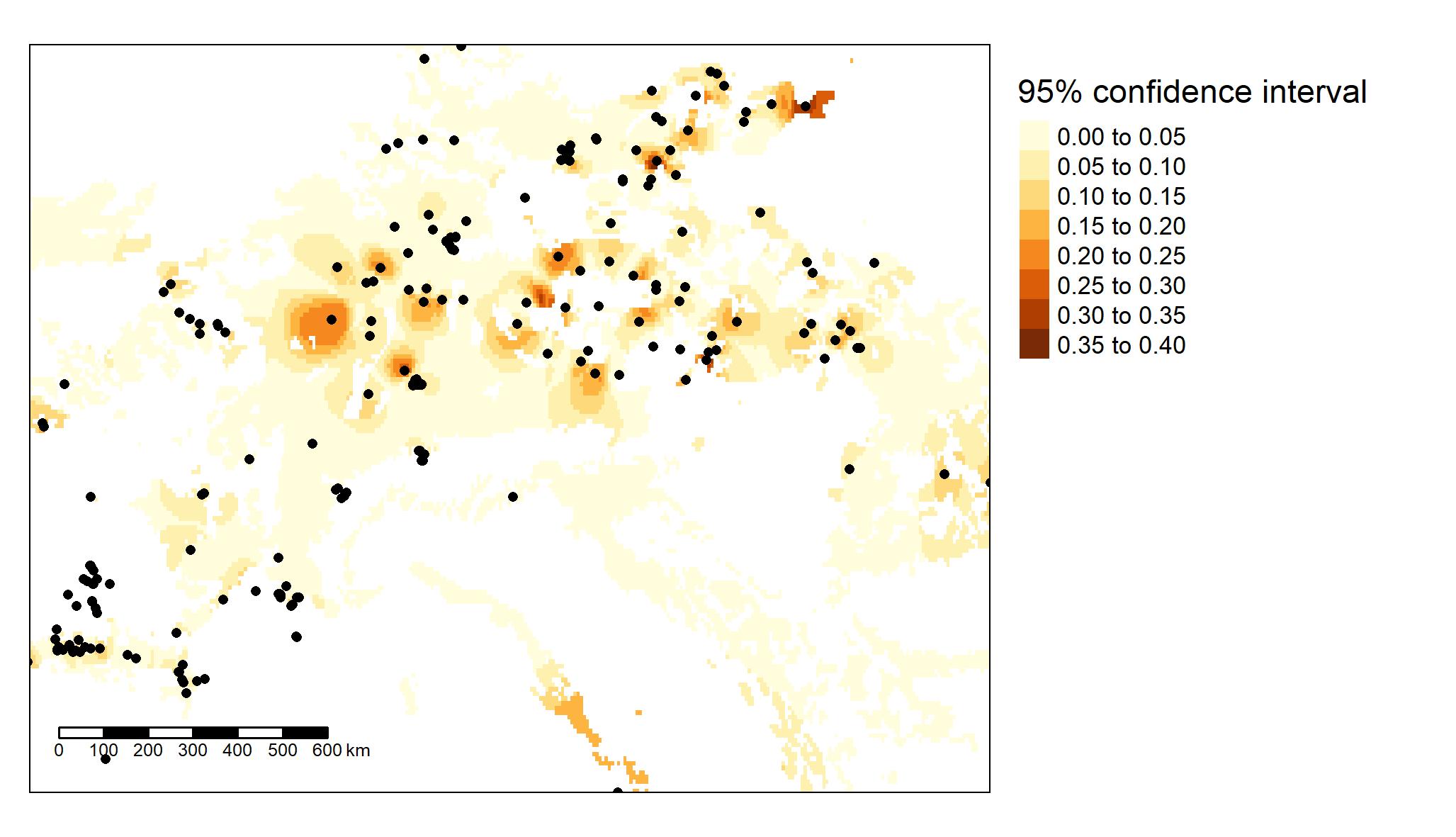

Supplement: Supplementary file 11 — Supplementary file11 (JPG 147 KB) [file 11295_2022_1577_MOESM11_ESM.jpg]
