# Supplementary material for: A novel synthesis of two decades of microsatellite studies on European beech reveals decreasing genetic diversity from glacial refugia
Source: Tree Genet Genomes. 2022 Dec 12;19(1):3. doi: 10.1007/s11295-022-01577-4 (PMC9744708; doi:10.1007/s11295-022-01577-4)
Supplement: Supplementary file 12 — Supplementary file12 (DOCX 114 KB) [file 11295_2022_1577_MOESM12_ESM.docx]

# Supplementary material

**Title**: A novel synthesis of marker-based studies on European beech across the species range reveals decreasing genetic diversity from glacial refugia.

**Article type**: Review

**Authors**: Camilla Stefanini (ORCID: 0000-0002-9156-5351)^1,a^, Katalin Csilléry (ORCID: 0000-0003-0039-9296)^2^, Bartosz Ulaszewski (ORCID: 0000-0003-2981-1205)^3^, Jarosław Burczyk (ORCID: 0000-0002-6899-2523)^3^, Michael E. Schaepman (ORCID: 0000-0002-9627-9565)^1^, Meredith C. Schuman (ORCID: 0000-0003-3159-3534)^1,4^

**Author affiliations**:

^1^Remote Sensing Laboratories, Department of Geography, University of Zurich, Winterthurerstrasse 190, 8057 Zurich, Switzerland

^2^Biodiversity and Conservation Biology Unit, Swiss Federal Research Institute WSL, Zürcherstrasse 111, 8903 Birmensdorf, Switzerland

^3^Department of Genetics, Faculty of Biological Sciences, Kazimierz Wielki University, Chodkiewicza 30, 85-064 Bydgoszcz, Poland

^4^Department of Chemistry, University of Zurich, Winterthurerstrasse 190, 8057 Zurich, Switzerland

^a^Current address:

**Author for correspondence**: Camilla Stefanini, [camstefa@gmail.com](mailto:camstefa@gmail.com)

**Table S1.** Summary of the data included in the literature review. Asterisks denote the total number of analysed trees.

| **Article** | **Country of sampled populations** | **Molecular marker** | **Number of loci** | **Biological sample** | **Tree age (years)** | **Plot size (km²)** | **Number of populations** | **Sample size** | **Genetic metrics** |
| --- | --- | --- | --- | --- | --- | --- | --- | --- | --- |
| Jump, Hunt, Martínez-Izquierdo, & Peñuelas, 2006 | Spain | AFLP | 254 | buds | < 40 | 2 | 3 | 70 | % P, *H*, SI, Fst, |
| Pluess and Weber, 2012 | Switzerland | AFLP | 517 | leaves | 120 | NA | 6 | 39 to 41 | % P, *He* |
| Jump et al., 2012 | Spain | AFLP | 317 | leaves | 80 | NA | 4 | 150 | *He* |
| Demesure, Comps, & Petit, 1996 | France, Spain, Belgium, the Netherland, Germany, Italy, Austria, Slovenia, Croatia, Czech Republic, Poland, Bulgaria, Romania, Sweden, Ukraine, Crimea | cpDNA microsatellites | 8 | buds | NA | 30 - 40 | 74 | 4.7 average (399*) | *Hs*, Ct, Gst |
| Hatziskakis S. et al., 2009 | Greece | cpDNA microsatellites | 13 (haplotypes) | leaves | NA | NA | 40 | 5 | *Ht, Hs*, Gst, Nst |
| Vettori et al., 2004 | Italy | cpDNA microsatellites PCR-RFLP | 4 | buds | NA | NA | 67 | 5 (355*) | No polymorphic fragments, *Ht, Hs,* Gst, Ct (na) |
| Magri et al., 2006 | across Europe (exact location NA) | cpDNA PCR-RFLP cpDNA microsatellites isozymes | 2  3  16 | buds | NA | NA | 352 (PCR/RFLP) 468 (isozymes) | 5 | *Vt, Vs*, Nst |
| Emiliani et al., 2004 | Italy | cpDNA-PCR/RFLP RAPD | 5 (haplotypes)  93 (RAPD) | buds | NA | NA | 30 | 5 (RFLP)  30 (RAPD) | *Hs, Ht*_,_ Gst, Ct (na) |
| Thiebaut Ph. Vernet, 1982 | France | isozymes | 2 | buds | NA | NA | 13 | (660-676*) | *all fr, v,* SI |
| Comps et al., 1990 | Poland, Italy, Corsica, Croatia, Romania, Bulgaria, Serbia, Czechoslovakia | isozymes | 6 | buds | NA | NA | 140 | 50 | *all fr, Ht, Hs*, Fis, Fst, Fit |
| Belletti et al., 1996 | Italy | isozymes | 8 | buds | NA | 100 - 120 | 11 | 80 | *He*, A/L, % P, *all fr, Ht,* *Hs*, Dst, Gst |
| Hazler et al., 1997 | Bulgaria, Macedonia, Slovenia, Croatia | isozymes | 11 | buds | NA | 30 - 40 | 38 | 50 | *all fr, Ht*, Fst, Fis, Fit |
| Gömöry, Hynek, & Paule, 1998 | Czech Republic, Slovakia, Poland | isozymes | 12 | buds | NA | NA | 20 | 50 | *all fr* |
| Sander, König, Rothe, Janßen, & Weisgerber, 2000 | Germany | isozymes | 13 | buds | 108 - 60 | 59 - 216 | 6 | 100 | *Ht, Hs*, Dst, Gst, Gst(%) |
| Comps et al., 2001 | across Europe (exact location NA) | isozymes | 12 | buds | NA | 30 - 40 | 389 | 47.4 average | *Ho, H*, A[g], Fis, *Hs, Ht*, Fst |
| Wang, 2003 | Germany | isozymes | 9 | seeds | 150 - 190 | 3.5 - 19.2 | 4 | 150, 180, 180, 190 | M, *V*, Vgam, *Ho,* δT |
| Wang, 2004 | Germany | isozymes | 9 | buds | 150 - 190 | 3.5 - 19.2 | 3 | 150, 180, 190 | A/L, % P, *Ho, He*, Fis, Fst |
| Dounavi et al., 2010 | Germany | isozymes | 9 | buds | 20 - 60, 140 | 2.5 - 13 | 2 | NA (all mapped trees) | Na, A/L, *v*, δT |
| Sulkowska M., 2010 | Poland | isozymes | 9 | buds | NA | 10 | 9 | NA | A/L, % P, *Ho, He* |
| Ballian et al., 2013 | Bosnia and Herzegovina | isozymes | 16 | seeds | NA | NA | 8 | 50 | Na, *all fr*, A/L, G/L, *Ho*, *He*, Fis |
| Leonardi et al., 2013 | Italy | isozymes | 11 | buds | NA | NA | 27 | 21.6 to 82.2 | Ar[20], Ar[40], % P, *He*, *Ho* |
| R. Pastorelli et al., 2002 | Italy | SSR | 7 | NA | NA | NA | 1 | 70 | Na, *Ho*, *He* |
| Vornam et al., 2004 | Germany | SSR | 4 | buds | 200 | NA | 1 | 99 | *Ho, He*, Ne, F |
| Buiteveld et al., 2007 | Germany, The Netherland, Austria, France, Italy | SSR | 4 | buds | 46 - 300 | 2.5 - 40 | 10 | 90 | Na, Ne, Arare, *Ho*, *He*,  Fst, Fis |
| Kraj et al., 2009 | Poland | SSR | 5 | leaves | 15 - 20 | NA | 3 | 90 | Na, Ne, *He*, Fst |
| Chybicki, Trojankiewicz, Oleksa, Dzialuk, & Burczyk, 2009 | Poland | SSR | 9 | leaves | NA | 98 - 114 | 2 | 241, 276 | Na, *He*, *Ho* |
| Nyàri 2010 | Germany, Hungary | SSR | 5 | buds and leaves | NA | NA | 2 | 76, 00 | Na, *Ho*, *He*, Fis |
| Lander et al., 2011 | France | SSR | 13 | leaves | NA | NA | 51 | (1932*) | Na, Ar, *Ho*, *He*, Fis |
| Lefevre et al., 2012 | France | SSR | 16 | buds and leaves | NA | NA | 4 | 40 to 45 | Na, Ar, *Ho*, *He*, Fis |
| Piotti et al., 2012 | Austria and France | SSR | 4 | buds | NA | NA | 4 | 376 to 427 | Na, *Ho*, *He*, NAF, Fis |
| DeLafontaine et al., 2013 | France and Spain | SSR | 16 | buds and leaves | NA | NA | 65 | 40 | Na, Ar, *Ho*, *He*, Fis |
| Pluess et al., 2013 | Switzerland | SSR | 19 | leaves | NA | NA | 6 | 10 | Na, Ar, *Ho*, *He*, Fis, Null |
| Gauzere et al., 2013 | France | SSR | 13 | leaves | NA | 13, 2.2, 8 | 3 | 137, 170, 194 | k, *Ho*, *He* |
| Rajendra et al. 2014 | Germany | SSR | 9 | leaves | 80-120 | 2 – 20 | 3 | 10 | At, Arare, Ne, *Hp*, *Hs*, Fis, Fst |
| Sjolund et al., 2015 | Germany, France, Italy | SSR | 11 | leaves or cambium | NA | 22.5 | 6 | 100 to 170 | Ar, Np, *Hs*, Fis |
| Bontemps et al., 2016 | France | SSR | 19 | leaves | NA | 8.3 | 1 | 149 | Na, *Ho*, *He*, Fis, NAF |
| Cvrkova et al., 2017 | Czech Republic | SSR | 12 | buds and leaves | 120 | NA | 13 | (390*) | Na, SI, *Ho*, *He*, Fst |
| Oddou-Muratorio et al., 2018 | France | SSR | 13 | leaves | NA | 13, 2.2, 8 | 3 | (722*) | Genotypes available |
| Nowakowska et al., 2011 | Poland | cpDNA microsatellites | 3 | leaves | NA | NA | 6 | 30 | *Ht*, *Hs*, Fst |
| Bilela et al., 2012 | Germany | SSR  isozymes | 5  16 | leaves, seedlings (SSR) | 77, 87 | NA | 2 | 80 | Fis, Dj (subpopulation differentiation) |
| Paffetti et al., 2012 | Italy | SSR  RAPD | 4  87 | buds | 207, 85 mean | 6.4, 2.7 | 2 | 100, 93 | Na, Arare, *He*, Null, Fit, Fis, Fst |
| Pluess et al., 2016 | Switzerland | SSR  SNPs | 12  144 | leaves | NA | 52 | 79 | 3 | Na, Ar, *Ho*, *He*, Fis, Null |
| Muller et al., 2018 | Switzerland, Germany | SSR  SNPs | 9  27 | leaves | NA | NA | 24 | 25 (CH), 100/200 (DE) | *He*, *Ho*, Gst, Ar, Fis |
| Cuervo-Alarcon et al., 2018 | Switzerland | SSR  SNPs | 13  76 | leaves | NA | NA | 12 | 25 | Ar, *Ho*, *He*, Fis, Fst, standardized Gst. Genotypes available |
| Seifert et al., 2012 | Germany | SNPs | 63 | leaves | NA | NA | 6 | 3 | Nhap, Dhap, nucleotide diversity |
| Lalague et al., 2014 | France | SNPs | 573 | leaves | NA | NA | 4 | (96*) | S (no segregating sites), θπ, Dhap |
| Capblancq et al., 2020 | France | SNPs | 7010 | leaves | 25 to 240 | NA | 36 | 570 | Genotypes available, *all fr*, F |
| Sandurska et al., 2009 | Poland | SSR | 20 | Leaves | > 218 | 55 | 1 | 333 | Na, Ne, Ar, *Ho*, *He*, Fis,  Null, Fst, Rst |
| Meger et al., 2019 | Poland | cpDNA SNPs | 8 | buds | NA | NA | 47 | 1/2 | Ne, Dhap, Nhap, Nehap, haplotypic richness |
| Kempf et al., 2016 | Poland | SSR | 10 | buds and leaves | 22 | NA | 12 | 46 | Na, A/L, Ne, *Ho*, *He*, Fis, Fst, Np |

% P – percentage of polymorphic loci, SI – Shannon diversity index, *He* – expected heterozygosity, *Ho* – observed heterozygosity, *Ht* or *Vt*– total genetic diversity, *Hs* or *Vs* – average genetic diversity within population, *H* – heterozygosity, *Vgam* – gametic diversity, M – allelic multiplicity, θπ – nucleotide diversity, *all fr* – allelic frequencies, A/L – mean number of alleles per locus, G/L – number of genotypes per locus, Na or *k* or At – number of alleles per locus, *v* – allelic diversity, Ne – effective number of alleles, Ar[*g*] – allelic richness for *g* gene copies, Arare – number of rare alleles (<5%), Np – number of private alleles, NAF or Null – null allele frequencies, Nhap – number of haplotypes, Dhap – haplotype diversity, Nehap – effective number of haplotypes, Fst – genetic differentiation, Gst and Nst– genetic differentiation among populations, Dst – genetic differentiation between subpopulations, δT – total genetic differentiation among populations, Fis – inbreeding coefficient, Fit – total genetic differentiation, Ct – contribution to total diversity, na – not available.

**Table S2.** Publications excluded from the review.

| **Reference** | **Reason for exclusion** |
| --- | --- |
| Scalfi et al., 2004 | Controlled cross |
| Comps et al., 1991 | Duplicated study |
| Csilléry et al., 2014 | Duplicated study |
| Gauzere et al., 2016 | Duplicated study |
| Piotti et al., 2013 | Duplicated study |
| Borghetti et al., 1993 | No field sampling |
| Cuguen et al., 1988 | No field sampling |
| Duputiè et al., 2015 | No field sampling |
| Frank et al., 2017 | No field sampling |
| Frank, Pluess et al., 2017 | No field sampling |
| G. Muller-Starck and R. Starke., 1993 | No field sampling |
| K. Kramer et al., 2010 | No field sampling |
| Krajmerovà et al., 2017 | No field sampling |
| Muller et al., 2015 | No field sampling |
| Muller et al., 2017 | No field sampling |
| Muller, 2013 PhD Thesis | No field sampling |
| Scalfi et al., 2004 | No field sampling |
| A. J. Hacket-Pain, Cavin, Friend, & Jump, 2016 | No genetic data |
| Aitor et al., 2017 | No genetic data |
| Andrew J. Hacket-Pain & Friend, 2017 | No genetic data |
| Aranda et al., 2017 | No genetic data |
| Bär et al., 2018 | No genetic data |
| Barbeta et al., 2011 | No genetic data |
| Barigah et al., 2013 | No genetic data |
| Bianchi et al., 2018 | No genetic data |
| Blondel, 2006 | No genetic data |
| Bolte et al., 2016 | No genetic data |
| Bontemps et al., 2017 | No genetic data |
| Bram K. et al., 2018 | No genetic data |
| Bresson et al., 2011 | No genetic data |
| Brinkmann et al., 2018 | No genetic data |
| Charru et al., 2017 | No genetic data |
| Chuine & Beaubien, 2001 | No genetic data |
| Ciais et al., 2005 | No genetic data |
| Conte et al., 2018 | No genetic data |
| Cordier et al., 2012 | No genetic data |
| Cuguen et al., 1988 | No genetic data |
| Dassot et al., 2012 | No genetic data |
| Defossez et al., 2015 | No genetic data |
| del Rio et al., 2017 | No genetic data |
| Denk et al., 2005 | No genetic data |
| Dittmar et al., 2003 | No genetic data |
| Drobyshev et al., 2014 | No genetic data |
| Durrant et al., 2016 | No genetic data |
| E Silva et al., 2012 | No genetic data |
| Ellegren & Galtier, 2016 | No genetic data |
| Evans et al., 2016 | No genetic data |
| Fotelli et al., 2001 | No genetic data |
| Fritz et al., 2008 | No genetic data |
| Gazol et al., 2019 | No genetic data |
| G. Piovesan et al., 2005 | No genetic data |
| Gailing, Vornam, Leinemann, & Finkeldey, 2009 | No genetic data |
| García-Plazaola & Becerril, 2000 | No genetic data |
| Gauzere et al., 2017 | No genetic data |
| Geilfus et al., 2017 | No genetic data |
| Gelmi-Cadusso et al., 2017 | No genetic data |
| Geßler et al., 2007 | No genetic data |
| Geßler et al., 2014 | No genetic data |
| Gianluca Piovesan & Schirone, 2000 | No genetic data |
| Gianluca Piovesan et al., 2008 | No genetic data |
| Gomory et al., 2015 | No genetic data |
| Gonzalez de Andres et al., 2018 | No genetic data |
| Gregory et al., 2018 | No genetic data |
| Hewitt, 1999 | No genetic data |
| Hewitt, 2011 | No genetic data |
| Heym et al., 2019 | No genetic data |
| Hoerling et al., 2012 | No genetic data |
| Hrivnak et al., 2016 | No genetic data |
| J. Bartolomé et al., 2000 | No genetic data |
| Jordi Bartolomé et al., 2005 | No genetic data |
| Jourdan et al., 2019 | No genetic data |
| Jump et al., 2007 | No genetic data |
| Jump, Hunt, & Pen̈uelas, 2006 | No genetic data |
| Keiner, Gruselle, Michalzik, Popp, & Frosch, 2015 | No genetic data |
| Knutzen et al., 2017 | No genetic data |
| Lars et al., 2019 | No genetic data |
| Latte et al., 2016 | No genetic data |
| Laurent et al., 2018 | No genetic data |
| Lespinas, Ludwig, & Heussner, 2010 | No genetic data |
| Llusia et al., 2013 | No genetic data |
| Losso et al., 2019 | No genetic data |
| Löw et al., 2006 | No genetic data |
| Magri D. 2008 | No genetic data |
| Mainiero & Kazda, 2006 | No genetic data |
| Manzanedo et al., 2018 | No genetic data |
| Menzel et al., 2015 | No genetic data |
| Metz et al., 2016 | No genetic data |
| Milleron et al., 2013 | No genetic data |
| Mishra et al., 2018 | No genetic data |
| Moning & Müller, 2009 | No genetic data |
| Mund et al., 2010 | No genetic data |
| Nielsen & Jørgensen, 2003 | No genetic data |
| O’Brien et al., 2017 | No genetic data |
| Oddou-Muratorio et al., 2010 | No genetic data |
| Officinale, 2011 | No genetic data |
| Ohlemüller et al., 2006 | No genetic data |
| Olga et al., 2017 | No genetic data |
| Paine et al., 2015 | No genetic data |
| Penuelas & Boada, 2003 | No genetic data |
| Peñuelas et al., 2007 | No genetic data |
| Peñuelas et al., 2008 | No genetic data |
| Peter et al., 2018 | No genetic data |
| Peuke et al., 2006 | No genetic data |
| Príncipe et al., 2017 | No genetic data |
| Prislan et al., 2019 | No genetic data |
| Raquel et al., 2020 | No genetic data |
| Rasmussen & Kollmann, 2004 | No genetic data |
| Robson et al., 2018 | No genetic data |
| Rose et al., 2009 | No genetic data |
| Saltré et al., 2013 | No genetic data |
| Seidel et al., 2019 | No genetic data |
| Stefan et al., 2019 | No genetic data |
| Stojnic et al., 2015 | No genetic data |
| Stojnić et al., 2018 | No genetic data |
| TEISSIER DU CROS et al., 1988 | No genetic data |
| Tognetti et al., 2019 | No genetic data |
| Van de Peer et al., 2017 | No genetic data |
| Varsamis et al., 2019 | No genetic data |
| Vitasse et al., 2010 | No genetic data |
| Vitasse et al., 2011 | No genetic data |
| Vitasse et al., 2019 | No genetic data |
| Vitasse et al., 2019(2) | No genetic data |
| Walter & Epperson, 2005 | No genetic data |
| Wortemann et al., 2011 | No genetic data |
| Widmer and Lexer, 2001 | No genetic data |
| Fang & Lechowicz, 2006 | No genetic data |
| Falk & Hempelmann, 2013 | No genetic data |
| Bolte et al., 2007 | No genetic data |
| Grundmann et al., 2011 | No genetic data |
| Michiels et al., 2009 | No genetic data |
| Cullotta et al., 2013 | No genetic data |
| Konnert et al., 1997 | Other language |
| Konnert, M. et al., 2000 | Other language |
| WD Maurer et al., 2008 | Other language |
| Barzdajan & Rzeznik, 2002 | Other language |
| Ammer et al., 2005 | Other language |
| Rennenberg et al., 2004 | Other language |
| Ahrens et al., 2018 | Review |
| Bolte et al., 2010 | Review |
| Giesecke et al., 2007 | Simulation |
| Hulsmann et al., 2016 | Simulation |
| Manzanedo et al., 2019 | Simulation |
| Oddou-Muratorio et al., 2014 | Simulation |
| Bragg, Supple, Andrew, & Borevitz, 2015 | Theoretical |
| Burghardt, Metcalf, Wilczek, Schmitt, & Donohue, 2015 | Theoretical |
| Matesanz & Valladares, 2014 | Theoretical |
| Rennison, Delmore, Samuk, Owens, & Miller, 2019 | Theoretical |
| Scotti et al., 2016 | Theoretical |

**Table S3.** Isozyme loci used for genotyping of *Fagus sylvatica* by studies included in the systematic review.

| **Isozyme system** | | **E.C. number** | | **Scored loci (Abbreviation)** | |
| --- | --- | --- | --- | --- | --- |
| 6-Phosphogluconate dehydrogenase | | 1.1.1.44 | | 6-Pgdh | |
| Acid phosphatase | | 3.1.3.2 | | Acp | |
| Aconitate hydratase | | 4.2.1.3 | | Aco | |
| Aspartate aminotransferase | | 2.6.1.1 | | Aat | |
| Diaphorase | | 1.6.4.3 | | Ld | |
| Glutamic-oxaloacetic transaminase | | 2.6.1.1 | | Got | |
| Isocitrate dehydrogenase | | 1.1.1.42 | | Idh | |
| Leucine aminopeptidase | | 3.4.11.1 | | Lap | |
| Malate dehydrogenase | | 1.1.1.37 | | Mdh | |
| Menadione reductase | | 1.6.99.2 | | Mnr | |
| Peroxidase | | 1.11.1.7 | | Per | |
| Phosphoglucomutase | | 2.7.5.1 | | Pgm | |
| Phosphoglucose isomerase | | 5.3.1.9 | | Pgi | |
| Shikimate dehydrogenase | | 1.1.1.25 | | Sad | |
| Succinate dehydrogenase | | 1.3.5.1 | | Sdh | |
| Superoxide dismutase | | 1.15.1.1 | | Sod | |
|  |  | |  | |  |

**Table S4.** Primers developed for *Fagus sylvatica* nuclear microsatellites which were used by the studies included in the systematic review. Reference indicates the study where the primers were developed.

| **Reference** | **Locus name** | **Repeat sequence** | **F Primer sequence (5'-3')** | **R Primer sequence** | **Accession no. / EST ID** | **Size range (bp)** | **Species developed for** |
| --- | --- | --- | --- | --- | --- | --- | --- |
| Pastorelli et al., 2003 | FS1-15 | (GA)26 | TCAAACCCAGTAAATTTCTCA | GCCTCAATGAACTCAAAAAC | AF528095 |  | *Fagus sylvatica* |
|  | FS1-25 | (GA)25 | GACCCATACCTCTCAGCTTC | AGAGATCATTGCAACCAAAC | AF528093 |  | *Fagus sylvatica* |
|  | FS1-03 | (GA)18 | CACAGCTTGACACATTCCAAC | TGGTAAAGCACTTTTTCCCACT | AF528090 |  | *Fagus sylvatica* |
|  | FS1-11 | (GA)15 | TGAATTCAATCATTTGACCATTC | GGAAGGGTGCTTCAATTTGG | AF528091 |  | *Fagus sylvatica* |
|  | FS3-04 | (GTC)5(GTT)3  (GTC)6 | AGATGCACCACTTCAAATTC | TCTCCTCAGCAACATACCTC | AF528092 |  | *Fagus sylvatica* |
|  | FS4-46 | (TGA)23 | GCAGTCCTCCACCATTACTA | TACAACAGCAGGCTATCCAT | AF528094 |  | *Fagus sylvatica* |
|  | FCM5 | (AG)10 | ACTGGGACAAAAAAACAAAA | GAAGGACCAAGGCACATAAA | from Tanaka |  | *Fagus sylvatica* |
| Pluess & Määttänen, 2013 | Fagsyl_000846 | (TTG) | GACCTGCTCCACGAACCTAC | GTCGAGACGGCTTTACATCG | NA | 118-127 | *Fagus sylvatica* |
|  | Fagsyl_000905 | (TGT) | GATCATAGCGCCGGAATTGG | GGTCCTCCTCCTGGTACAAC | NA | 149-171 | *Fagus sylvatica* |
|  | Fagsyl_001018 | (CA) | AATATCAGGGAGGCAGCACC | CGAGATGGACTTCTAAGTTTTATTTGC | NA | 107-126 | *Fagus sylvatica* |
|  | Fagsyl_001217 | (CA) | GGATGGGTTTTTGGCTCAGG | CGTTGTCATGCAGGAGTGTG | NA | 133-156 | *Fagus sylvatica* |
|  | Fagsyl_002140 | (ACA) | CGAAGCGACAAACCTCTCTG | TGGGAGAATACGTCCAAGGC | NA | 113-133 | *Fagus sylvatica* |
|  | Fagsyl_002929 | (TTG) | GCGGCGACTGGAATAATAGC | CAATCACACGCTGCACAAAC | NA | 152-204 | *Fagus sylvatica* |
|  | Fagsyl_004597 | (CA) | GGAGAAAACCTTCTGTCCTCAC | TGTAGAGAAACCTAGAGCTTGAC | NA | 96-121 | *Fagus sylvatica* |
|  | Fagsyl_003273 | (GT) | GGATCCACCTGGCACTTTTG | TGCAATATTACCCTGGGCTG | NA | 185-191 | *Fagus sylvatica* |
|  | Fagsyl_003994 | (GA) | ACAAAGGAATCGTGGAGCTG | ACACATTCTGCCTCAAAGTACC | NA | 110-136 | *Fagus sylvatica* |
|  | Fagsyl_003093 | (CA) | TCATCACCGAGACAAGGGAC | ATGGTGGTGTGGAAGCTAGG | NA | 148-205 | *Fagus sylvatica* |
|  | Fagsyl_003849 | (CT) | GCTTCGTTCTTCGGCATCTC | AATAGCACAAATAGCCCGCC | NA | 98-128 | *Fagus sylvatica* |
| **Reference** | **Locus name** | **Repeat sequence** | **F Primer sequence (5'-3')** | **R Primer sequence** | **Accession no. / EST ID** | **Size range (bp)** | **Species developed for** |
|  | Fagsyl_001563 | (TG) | TGATTTTGCCTATCAGGCTCC | CTGGCTCAAGAGACAAGAGG | NA | 113-125 | *Fagus sylvatica* |
|  | Fagsyl_004467 | (TGA) | GACCCACTTCCTTTACTGCC | TCATCATCACCACCGCTCAC | NA | 105-120 | *Fagus sylvatica* |
|  | Fagsyl_007038 | (TG) | ACCAGCAAATCCACGTATTAAC | GTGGGCTTGTGACTTGCTTC | NA | 152-166 | *Fagus sylvatica* |
|  | Fagsyl_003282 | (CA) | GTTAGCTTAACTCATCTGGGCG | TTTCAGTTGCCTGAGAACCC | NA | 204-215 | *Fagus sylvatica* |
|  | Fagsyl_000909 | (AC) | TCACTTGATGCCACATGCAC | CGCCCATCGCTTGAAGTAAG | NA | 150-166 | *Fagus sylvatica* |
|  | Fagsyl_006075 | (TG) | TGGATGCAATCCGTGAACTG | TGGTCTCTTCCTCGTCCAAG | NA | 156-269 | *Fagus sylvatica* |
|  | Fagsyl_006608 | (TC) | TCGTTCTTTTTCAAATATGCAGGTG | TGCACAAGAGCATGTGTACG | NA | 99-127 | *Fagus sylvatica* |
| Lefèvre et al., 2012 | csolfagus_31 | (AG)12 | TCTATTGACACAAGAATAAGAACACC | CTTGGCAAGAAAAGGGGATT | NA | 104-126 | NA |
|  | sfc_1143 | (AG)21 | TGGCATCCTACTGTAATTTGA | ATTCCACCCACCATCTGTC C | NA | 112–130 | NA |
|  | csolfagus_05 | (GA)10 | GGTTTCTAGCAAAATTGGCATT | CCCAAAAGGCCCTACTACAA | NA | 167-179 | NA |
|  | FS1_15 | (GA)26 | TCAAACCCAGTAAATTTCTCA | GCCTCAATGAACTCAAAAAC | NA | 95–137 | NA |
|  | sfc_0036 | (TC)23 | CATGCTTGACTGACTGTAAGTTC | TCCAGGCCTAAAAACATTTATAG | NA | 94–112 | NA |
|  | csolfagus_06 | (AG)13 | GTTGTTGCTCACAGCAGTCG | ACGCTTGGTCTTCTTGCACT | NA | 203-221 | NA |
|  | csolfagus_19 | (TC)13 | TGCCCATGAGGTTTGTATCA | GCCGAATAACCCAGAAAACA | NA | 154–182 | NA |
|  | csolfagus_29 | (CT)11 | CACAACCTGCATTCCCTTTC | GTTTGGCACTTTGGCTTGTT | NA | 132-148 | NA |
|  | EEU75_A_0 | (CT)10 | TTCCAAACCAACCCTTTATCC | GACGGAGATTGAGGAAGAACA | NA | 91–111 | NA |
|  | DUKCT_A_0 | (AC)14 | GCCTCTCGCAGCTCCTATAA | GATCTAATGTGGGTTTGGTTTTG | NA | 75-95 | NA |
|  | EJV8T_A_0 | (TC)10 | CCTGTTCTCACACTTGGGTCTA | TGCATTACAAAGCCTGAAACA | NA | 143–155 | NA |
|  | EMILY_A_0 | (GA)11 | GACCCCAAGGTTACAGTGCT | CGTACAATTGCACCCACATC | NA | 142–152 | NA |
|  | ERHBI_A_0 | (AG)9 | TGCAACAACTTAGCACTTTGA | GCGTGTGGCTTATCCAAAAT | NA | 159–167 | NA |
|  | DZ447_A_0 | (TC)10 | GGTGCAATACTTCACTTTAGGACA | ATAGGAGTGGGACGGCTAGG | NA | 186–194 | NA |
|  | concat14_A_0 | (TC)9 | TGAAGAAATTCACAACCCAACA | GGGTTGTTTACGATGGTGGA | NA | 173–197 | NA |
|  | DE576_A_0 | (CAA)10 | TCTCCTTAGATCCACAATCACA | AGCTCTTCATTGCTCAGAACG | NA | 211–232 | NA |
| **Reference** | **Locus name** | **Repeat sequence** | **F Primer sequence (5'-3')** | **R Primer sequence** | **Accession no. / EST ID** | **Size range (bp)** | **Species developed for** |
| Vornam et al., 2004 | mfc2 | (CT)27 | ACAACTAATCCCACCAGTCC | GGCCTAAAGCGACAACTCTA | NA | Multi-locus | NA |
|  | mfc3 | (ATT)9 | TAATGGGATGGAGGGTGGAT | GGAAGACAAACAATGGAAAG | NA | No amplification | NA |
|  | mfc4 | (CT)6 | ATMAGCAACTGAATAAAGA | GTCAAGAGAGGGAAGAGAGT | NA | Multi-locus | NA |
|  | mfc5 | (AG)10 | ACTGGGACAAAAAAACAAAA | GAAGGACCAAGGCACATAAA | NA | 277–329 | *Fagus crenata* |
|  | mfc7 | (GA)9 | AAAATACACTGCCCCAAAA | CAGGTTTTGGTTTCTTACAC | NA | Multi-locus | NA |
|  | mfc9-2 | (GA)19 | TTCCCTCCTCTTCTCAAAT | TTATACTTCCTCTCTCATCCC | NA | 182–200 | *Fagus crenata* |
|  | mfc11 | (AG)10 | ACAGATAAAAACAGAAGCCA | TTTGGTTTTGTTGAGTTTAG | NA | 312–331 | *Fagus crenata* |
|  | mfs11 | (AG)10 | GGGGGTGGTTTCAAGTTTC | GAAGCCAATTATCACACCAAAAC | NA | 125–144 | NA |
|  | mfc12 | (AGG)(AG)6(AGG)  (AG)7(AGG) | ACACCTCACAATCCACGAAA | CCCAATAACTAAGAATACCA | NA | No amplification | NA |
|  | mfc13 | (CT)4(GT)2(CA)2 | GAGAGCAGGATGGGATGAAA | ACAGCACCTCTCCTTCTCTT | NA | No amplification | NA |
| Asuka et al., 2004 | sfc0007–1 | (AAAT)4 | GCATTCAATTAGAACAAGAGG | TCAATGTTTGCGACAATAAC | AJ586309 | 136–170 | *Fagus crenata* |
|  | sfc0007–2 | (AG)24 | TGTCGCAAACATTGACAAGG | GTGGATGTGAGGTCGTTGG | AJ586309 | 149–157 | *Fagus crenata* |
|  | sfc0018 | (AG)17 | GAAGCAGAGCATTGTATTGG | CATCTGTTTCAGTTCTGTAAAGG | AJ586310 | 161–191 | *Fagus crenata* |
|  | sfc0036 | (TC)23 | CATGCTTGACTGACTGTAAGTTC | TCCAGGCCTAAAAACATTTATAG | AJ586311 | 96–142 | *Fagus crenata* |
|  | sfc0109 | (GA)27 | TTGGTGGTCAACATCAC | TGACCATTAAGTCAACAATC | AJ586312 | 93–175 | *Fagus crenata* |
|  | sfc0146 | (TC)17 | TCGATTTCAGACGTGATG | TCCGCCAATTTGGTATG | AJ586313 | 130–202 | *Fagus crenata* |
|  | sfc0161 | (AG)22 | AAGCTCCACGATTCATTC | GCTGGAGTTGCTCTAAGTC | AJ586314 | 77–165 | *Fagus crenata* |
|  | sfc0195–2 | (TC)7 | CCAGCCTCTCGTCTATTATC | AATGGAATGCTTGTTCAAC | AJ586315 | 175–187 | *Fagus crenata* |
|  | sfc0289–1 | (AG)8 | GGAAAGCTTGGTACTATTAGAG | AAGAGAAGCTTAGTCATGTACAC | AJ586316 | 142–186 | *Fagus crenata* |
|  | sfc0305 | (GA)24 | CCAATGGACTTGTTATACCAATC | GCACCAGTTGCTTACAGAATAG | AJ586317 | 159–203 | *Fagus crenata* |
|  | sfc360–2 | (AG)6 | ATGCTTTGCTGTTCAAGATG | TTTGCATAAACTCACTCTCAGTC | AJ586318 | 105–109 | *Fagus crenata* |
| **Reference** | **Locus name** | **Repeat sequence** | **F Primer sequence (5'-3')** | **R Primer sequence** | **Accession no. / EST ID** | **Size range (bp)** | **Species developed for** |
|  | sfc0378 | (AG)14 | CCTAAAATTCAGTGATGATTATG | TGGCTTTGAGTCTGAGATG | AJ586319 | 223–249 | *Fagus crenata* |
|  | sfc0488 | (GA)15 | TCTCGATTTATAGTGTTTCTG | CATCCTTGTACTTCTCTAACAG | AJ586320 | 129–153 | *Fagus crenata* |
|  | sfc1063 | (CT)13 | TTTCCAACTACAACTTCATTG | AGTGCTCGCATCGTATG | AJ586321 | 188–222 | *Fagus crenata* |
|  | sfc1105 | (TC)31 | TCGTCTCTTCCGTCATCAC | CAGCGTATACCTAATCAATTCC | AJ586322 | 120–180 | *Fagus crenata* |
|  | sfc1143 | (AG)21 | TGGCATCCTACTGTAATTTGAC | ATTCCACCCACCATCTGTC | AJ586323 | 96-136 | *Fagus crenata* |
| Tanaka et al., 1999 | mfc2 | (CT)27 | ACAACTAATCCCACCAGTCC | GGCCTAAAGCGACAACTCTA | NA | 134-219 | *Fagus crenata* |
|  | mfc3 | (ATT)9 | TAATGGGATGGAGGGTGGAT | GGAAGACAAACAATGGAAAG | NA | 201-211 | *Fagus crenata* |
|  | mfc4 | (CT)6 | ATAAAGCAACTGAATAAAGA | GTCAAGAGAGGGAAGAGAGT | NA | 190-198 | *Fagus crenata* |
|  | mfc5 | (AG)10 | ACTGGGACAAAAAAACAAAA | GAAGGACCAAGGCACATAAA | NA | 256-320 | *Fagus crenata* |
|  | mfc7 | (GA)9 | AAAATACACTGCCCCCAAAA | CAGGTTTTGGTTTCTTACAC | NA | 103-135 | *Fagus crenata* |
|  | mfc9-2 | (GA)19 | TTCCCTCCTCTTCTCTAAAT | TTATACTTCCTCTCTCATCCC | NA | 164-186 | *Fagus crenata* |
|  | mfc11 | (AG)10 | ACAGATAAAAACAGAAGCCA | TTTGGTTTTGTTGAGTTTAG | NA | 313-337 | *Fagus crenata* |
|  | mfc12 | (AGG)(AG)6(AGG)  (AG)7(AGG) | ACACCTCACAATCCACGAAA | CCCAATAACTAAGAATACCA | NA | 280-327 | *Fagus crenata* |
|  | mfc13 | (CT)4(GT)2(CA)2 | GAGAGCAGGATGGGATGAAA | ACAGCACCTCTCCTTCTCTT | NA | 315-337 | *Fagus crenata* |
| Marinoni et al., 2003; | CsCAT14 | (CA)22 | CGAGGTTGTTGTTCATCATTAC | GATCTCAAGTCAAAAGGTGTC | NA | 164 | *Castanea sativa* |
| Durand et al., 2010 | FIR004 | (CT)18 | TCTCTCTCAGGGCAGCTTCT | AACCAAACTCAGATCCAGATTCA | LG0AAC23YD19RM1.SCF, G0AAC4YC16RM1.SCF | 160 | *Quercus robur* |
|  | FIR065 | (CTT)9 | ATTCCCATGCATCAAAATCC | TCCTTCAGTTTGAGAGCTCCTT | LG0AAA18YN08RM1.SCF, LG0AAC17YE02RM1.SCF, LG0AAC6YM22RM1.SCF | 190 | *Quercus robur* |
|  | GOT066 | (GAA)10 | TCCCTAGATGATGGGGATGA | TTTTACGTCGGCCAACTTTT | LG0AAA18YP04RM1.SCF stackPACK526_CT_3327 start 618 8.00 GAA end 641 | 238 | *Quercus robur* |
| **Reference** | **Locus name** | **Repeat sequence** | **F Primer sequence (5'-3')** | **R Primer sequence** | **Accession no. / EST ID** | **Size range (bp)** | **Species developed for** |
| Ueno et al., 2009 | Fc3 (FcC00468) | (TAT)3TCTTGTGACG  TTTTGAAGTG(AG)11 | CACACACGCACAAAACACACAAA | TGGGTGACATTCGGAGAACACTT | Fc_TUM08_04I22 DC65269 | NA | *Fagus crenata* |
|  | Fc5 (FcC00730) | (GCT)6 | ATGGGGTCTTATCACGGTTTTGC | GTTTTTCAGCGATTTCCCCAAAG | Fc_TUM08_12E15 DC655417 | NA | *Fagus crenata* |
|  | Fc6 (FcC00927) | (TC)3CCAAAAAAAA  AA(AC)3(TC)16 | CCTGCCGCTCAATTATTAGTCCC | ACGAGTCAGCTGGAAAACACGAG | Fc_TUM08_06D23 DC653288 | NA | *Fagus crenata* |
|  | Fc9 (FcC03095) | (TC)17 | GATTCTCTGCAACTCTGCAAGGC | TCTGGGTACCTCGAACCAGTCAA | Fc_TUM08_15L23 DC656594 | NA | *Fagus crenata* |

1. **Table S5.** Summary of the systematic review. Genetic diversity metrics are recorded for different molecular markers; data are reported as minimum value, mean (standard deviation) and maximum value; *N_pub_ –* number of publications*, N_pop_* – number of populations, *He* – expected heterozygosity (reported for nuclear markers), *H* – haplotypic diversity (reported for chloroplast markers), *Ar* – allelic richness (nuclear), *Rh* – haplotypic richness (chloroplast). In brackets the country where the data was recorded.

| **Molecular marker** | | **N_pub_** | **N_pop_** | **Genetic diversity** | | | | | |
| --- | --- | --- | --- | --- | --- | --- | --- | --- | --- |
|  |  |  |  | **He** | | | **Ar** | | |
|  |  |  |  | **Min** | **Mean (SD)** | **Max** | **Min** | **Mean (SD)** | **Max** |
| nuclear | AFLP | 3 | 12 | 0.172  (ES) | 0.229 (0.023) | 0.25  (CH) | NA | NA | NA |
|  | RAPD | 2 | 31 | 0.246  (IT) | 0.251 (0.008) | 0.257  (IT) | NA | NA | NA |
|  | microsatellites | 24 | 378 | 0.5135  (PL) | 0.681 (0.061) | 0.87  (DE) | 4.2  (FR) | 6.57  (2.352) | 18.2 (IT) |
|  | SNP | 6 | 161 | 0.226  (DE) | 0.26 (0.096) | 0.335  (CH) | NA | NA | NA |
|  | isozymes | 14 | 641 | 0.12  (IT) | 0.297 (0.041) | 0.3615  (HR) | 0.47  (IT) | 2.755 (0.053) | 2.9  (NA) |
|  |  |  |  | **H** | | | **Rh** | | |
|  |  |  |  | **Min** | **Mean (SD)** | **Max** | **Min** | **Mean (SD)** | **Max** |
| chloroplast | PCR-RFLP | 3 | 449 | 0.57  (IT) | 0.695 (0.12) | 0.82  (IT) | NA | NA | NA |
|  | RAPD | 1 | 30 | NA | NA | NA | NA | NA | NA |
|  | microsatellites | 5 | 539 | 0.24 (NA) | 0.483 (0.21) | 0.83  (EL) | NA | NA | NA |
|  | SNP | 1 | 47 | 0.937 (DE) | 0.937  (0) | 0.937  (DE) | 24 | NA | 24 |

## Analysis of SNP data (Supplementary Methods)

SNP-based genetic diversity was reported as *He* (Cuervo-Alarcon et al. 2018; Müller et al. 2018; Pluess et al. 2016) or nucleotide diversity (Lalagüe et al. 2014 and Seifert, Vornam, and Finkeldey 2012) on a per-population basis or averaged across all analyzed stands. Since individual genotype SNP data were not available for these publications, no additional computation or standardization was possible. Average *He* was estimated for the SNP dataset kindly provided by Capblancq, Morin, et al. (2020) for each individual by using VCFtools version 0.1.16 (command "--*het*", Danecek et al., 2011)*,* and successively averaged on a per-population basis using RStudio.

- 1. Genetic diversity derived from different marker systems (Supplementary results)

For the sake of completeness, we here report the summarized results from the systematic review. As these can be misleading (we did not attempt any scaling or other approach to make studies more comparable), we report them here in the supplementary and advise readers to treat these as only a very rough characterization of available knowledge.

*Ar* and *He* were reported in 14 and 40 publications, respectively, although six of these publications did not indicate the sample size used for the calculation or whether rarefaction or bootstrapping was used to account for effects of sample size. The highest *Ar* value based on isozymes (2.9) was reported by Comps et al. 2001 as a mean value across populations in their study. The mean value across the studies considered in this review is 2.76 ± 0.53. For nuclear microsatellites, the population with the highest *Ar* value (18.2) was located in Abruzzo according to Buiteveld et al. 2007, and the mean *Ar* across studies was 6.57 ± 2.35. We note that these values are not normalized and were not calculated with the same sample size, thus not suitable for comparison between studies. Mean *He* was 0.43 ± 0.019 for nuclear markers; 0.87 was the highest value reported by Buiteveld et al., 2007 for the German population Flecken-Zechlin 2. For the populations analyzed with two different types of markers, significant correlations between *He* estimates based on different markers was observed only in one case out of four (Müller et al., 2018; Spearman correlation *r_s_* = 0.446, *p* = 0.014).

For chloroplast markers, 0.937 was the highest value for haplotypic diversity, recorded by Meger et al. 2019 across Czech beech populations, while the overall mean across populations was 0.58 ± 0.038 (Table S5).

SNP-based genetic diversity was expressed either as nucleotide diversity (Lalagüe et al. 2014; Seifert, Vornam, and Finkeldey 2012, mean 0.58 ± 0.096) or observed and expected heterozygosity (Cuervo-Alarcon et al., 2018; Müller et al., 2018; Pluess et al., 2016, mean 0.26 ± 0.019) on a per-population basis or as mean across all the populations. These studies mainly covered the areas of the French Alps and Switzerland (Pritchard et al. 2000; Falush et al. 2003; Bradbury et al. 2007; Antao et al. 2008; Foll and Gaggiotti 2008; Excoffier et al. 2009b; Günther and Coop 2013; Frichot et al. 2015), while a few stands were investigated in Germany and in Sweden (Fig. 2d).

Genetic structure (Supplementary results)

The average global *Fst* value (Wright’s fixation index) and the *Fis* index (inbreeding coefficient) were calculated and reported in 30 and in 22 studies, respectively. Mean values for Fst were 0.78 ± 0.07 for cpDNA markers and 0.035 ± 0.02 for nuclear markers, while overall the mean inbreeding coefficient across all the studies was 0.045 ± 0.068, with three studies (Buiteveld et al. 2007; Paffetti et al. 2012; Piotti et al. 2012) reporting a value higher than 0.2.

**Figure S1.** Distribution of allelic frequencies from nuclear microsatellite studies compiled into a metadataset.

**Figure S2.** Loci included in the genotyping kits used in the studies based on nuclear microsatellites considered in the systematic review. Light orange denotes loci used in the study, and dark blue denotes loci not used. Lateral bar indicates the clustering of the studies.

**Figure S3.** *He* of *Fagus sylvatica* populations determined by nuclear microsatellite loci before (blue) and after scaling (yellow).

**Figure S4**. Spearman correlation scatter plots (linear regression as blue line with confidence interval as gray area) between latitude (left) and Distance from origin (right) and the raw and scaled He dataset from Ulaszewski et al., 2021 by considering the whole dataset, only the loci developed by Pastorelli, Vendramin and Asuka et al., only the loci developed by Lefevre et al. 2012 and only the loci developed by Ueno et al. 2009 for *F. crenata*. Spearman correlation coefficient and associated p-values are reported inside the correlation plot.

**Figure S5.** Scatterplot of the predicted vs. observed He values for the interpolation model, the solid diagonal line represents the 1:1 slope, the red dashed line is the linear fit to the points.

**Figure S6.** 95% confidence interval map of the IDW interpolation model (power parameter = 5).

**Figure S7**. Scatterplot of the predicted vs. observed *He* values for the interpolation model, the solid diagonal line represents the 1:1 slope, the red dashed line is the linear fit to the points.

**Figure S8**. 95% confidence interval map of the IDW interpolation model (power parameter = 5).
